# Lipid Signaling via Pkh1/2 Regulates Fungal CO_2_ Sensing through the Kinase Sch9

**DOI:** 10.1128/mBio.02211-16

**Published:** 2017-01-31

**Authors:** Susann Pohlers, Ronny Martin, Thomas Krüger, Daniela Hellwig, Frank Hänel, Olaf Kniemeyer, Hans Peter Saluz, Patrick Van Dijck, Joachim F. Ernst, Axel Brakhage, Fritz A. Mühlschlegel, Oliver Kurzai

**Affiliations:** a Septomics Research Center, Leibniz Institute for Natural Product Research and Infection Biology-Hans Knöll Institute (HKI), Jena, Germany; b Friedrich Schiller University Jena, Jena, Germany; c Department of Molecular and Applied Microbiology, Leibniz Institute for Natural Product Research and Infection Biology-Hans Knöll Institute (HKI), Jena, Germany; d Department of Cell and Molecular Biology, Leibniz Institute for Natural Product Research and Infection Biology-Hans Knöll Institute (HKI), Jena, Germany; e VIB Department of Molecular Microbiology, KU Leuven, Leuven, Belgium; f Laboratory of Molecular Cell Biology, KU Leuven, Leuven, Belgium; g Department Biologie, Molekulare Mykologie, Heinrich-Heine-Universität, Düsseldorf, Germany; h Kent Fungal Group, School of Biosciences, University of Kent, Canterbury, United Kingdom; i Clinical Microbiology Service, East Kent Hospitals University NHS Foundation Trust, The William Harvey Hospital, Ashford, United Kingdom; j Center for Sepsis Control and Care, University Hospital Jena, Jena, Germany; University of Texas Health Science Center

## Abstract

Adaptation to alternating CO_2_ concentrations is crucial for all organisms. Carbonic anhydrases—metalloenzymes that have been found in all domains of life—enable fixation of scarce CO_2_ by accelerating its conversion to bicarbonate and ensure maintenance of cellular metabolism. In fungi and other eukaryotes, the carbonic anhydrase Nce103 has been shown to be essential for growth in air (~0.04% CO_2_). Expression of* NCE103* is regulated in response to CO_2_ availability. In *Saccharomyces cerevisiae*, *NCE103* is activated by the transcription factor ScCst6, and in *Candida albicans* and *Candida glabrata*, it is activated by its homologues CaRca1 and CgRca1, respectively. To identify the kinase controlling Cst6/Rca1, we screened an *S. cerevisiae* kinase/phosphatase mutant library for the ability to regulate *NCE103* in a CO_2_-dependent manner. We identified ScSch9 as a potential ScCst6-specific kinase, as the *sch9*Δ mutant strain showed deregulated *NCE103* expression on the RNA and protein levels. Immunoprecipitation revealed the binding capabilities of both proteins, and detection of ScCst6 phosphorylation by ScSch9 *in vitro* confirmed Sch9 as the Cst6 kinase. We could show that CO_2_-dependent activation of Sch9, which is part of a kinase cascade, is mediated by lipid/Pkh1/2 signaling but not TORC1. Finally, we tested conservation of the identified regulatory cascade in the pathogenic yeast species *C. albicans* and *C. glabrata*. Deletion of *SCH9* homologues of both species impaired CO_2_-dependent regulation of *NCE103* expression, which indicates a conservation of the CO_2_ adaptation mechanism among yeasts. Thus, Sch9 is a Cst6/Rca1 kinase that links CO_2_ adaptation to lipid signaling via Pkh1/2 in fungi.

## IMPORTANCE

All living organisms have to cope with alternating CO_2_ concentrations as CO_2_ levels range from very low in the atmosphere (0.04%) to high (5% and more) in other niches, including the human body. In fungi, CO_2_ is sensed via two pathways. The first regulates virulence in pathogenic yeast by direct activation of adenylyl cyclase. The second pathway, although playing a fundamental role in fungal metabolism, is much less understood. Here the transcription factor Cst6/Rca1 controls carbon homeostasis by regulating carbonic anhydrase expression. Upstream signaling in this pathway remains elusive. We identify Sch9 as the kinase controlling Cst6/Rca1 activity in yeast and demonstrate that this pathway is conserved in pathogenic yeast species, which highlights identified key players as potential pharmacological targets. Furthermore, we provide a direct link between adaptation to changing CO_2_ conditions and lipid/Pkh1/2 signaling in yeast, thus establishing a new signaling cascade central to metabolic adaptation.

## INTRODUCTION

Carbon dioxide is key to life on earth. Besides being RuBisCO’s substrate in the carbon-fixing component of the Calvin cycle, it is the final product of cellular respiration (1). Consequently CO_2_ plays a decisive role as a signaling molecule, and its levels are sensed by organisms as diverse as bacteria, plants, fungi, nematodes, insects, fish, and mammals (2, 3). In fungi, CO_2_ impacts fundamental biological characteristics, including growth, morphology, and virulence (4). For pathogenic fungi, the ability to adapt to changing CO_2_ is particularly relevant. When surviving as commensals on skin, fungi are exposed to atmospheric low CO_2_ concentrations, but when they invade their host, CO_2_ concentrations can rise to 150-fold higher levels (5% or more) (5–7).

In fungi, CO_2_ is sensed via two distinct signaling pathways. In the first, HCO_3_^−^, which is in equilibrium with membrane-permeable CO_2_ via action of the catalytic enzyme carbonic anhydrase (CA), directly activates adenylyl cyclases to signal via downstream protein kinase A (PKA) (8). This pathway was shown to trigger the yeast-to-hyphae switch of *Candida albicans* and capsule biosynthesis of *Cryptococcus neoformans*, representing well-characterized virulence factors of these major human pathogens (9, 10).

The second pathway is independent of adenylyl cyclase and much less characterized. This pathway directly affects cellular CO_2_ fixation and HCO_3_^−^ homeostasis and thus central metabolism. One readout of this pathway is regulation of expression of the β-class fungal CA Nce103p in response to environmental CO_2_ (11, 12). CAs are ubiquitous zinc-containing metalloenzymes that accelerate the CO_2_ to HCO_3_^−^ interconversion and thus impact carboxylation reactions and pH homeostasis (13–15).

Notably yeast CA expression is strongly induced under CO_2_-limiting atmospheric conditions and downregulated in elevated CO_2_ (11). The overarching significance of this second CO_2_-sensing pathway for fungal biology is emphasized by the fact that deletion of yeast CA abolishes growth in low CO_2_ (16).

CO_2_-dependent regulation of CA is conserved in *Saccharomyces cerevisiae*, *C. albicans*, and *Candida glabrata*. Some insight into the signaling events mediating CO_2_-dependent regulation of CA expression in yeast was recently gained by identification of the transcriptional activator regulator of carbonic anhydrase 1 (Rca1). Loss of Rca1 in the pathogenic yeasts *C. albicans* and *C. glabrata* and loss of the Rca1 orthologue Cst6 in *S. cerevisiae* led to a common phenotype displaying lack of CA induction in low CO_2_ (17, 18). Rca1 and Cst6 belong to the ATF/CREB transcription factor family and have a C-terminal basic leucine zipper domain (19). Comparison of the protein sequence of yeast Rca1/Cst6 revealed 3 conserved serine phosphorylation sites, and loss of serine 124 in the *C. albicans* orthologue abolished CO_2_-dependent regulation of downstream CA (17). This supports our hypothesis that Rca1/Cst6 phosphorylation is a critical step in yeast CO_2_ sensing.

To unravel this novel CO_2_-sensing pathway, we used *S. cerevisiae* as a model. Using high-throughput kinase/phosphatase mutant library screening, we identified Sch9 as the Cst6-specific kinase candidate. We confirmed direct interaction and phosphorylation of Cst6 by the Sch9 kinase and show that Cst6/Rca1 signaling is conserved in *C. albicans* and *C. glabrata* CO_2_ sensing. Finally, we provide evidence that Sch9 links CO_2_ adaptation to lipid signaling via Pkh1/2.

## RESULTS

### High-throughput screening identifies potential Cst6 kinases.

**FIG 1:**
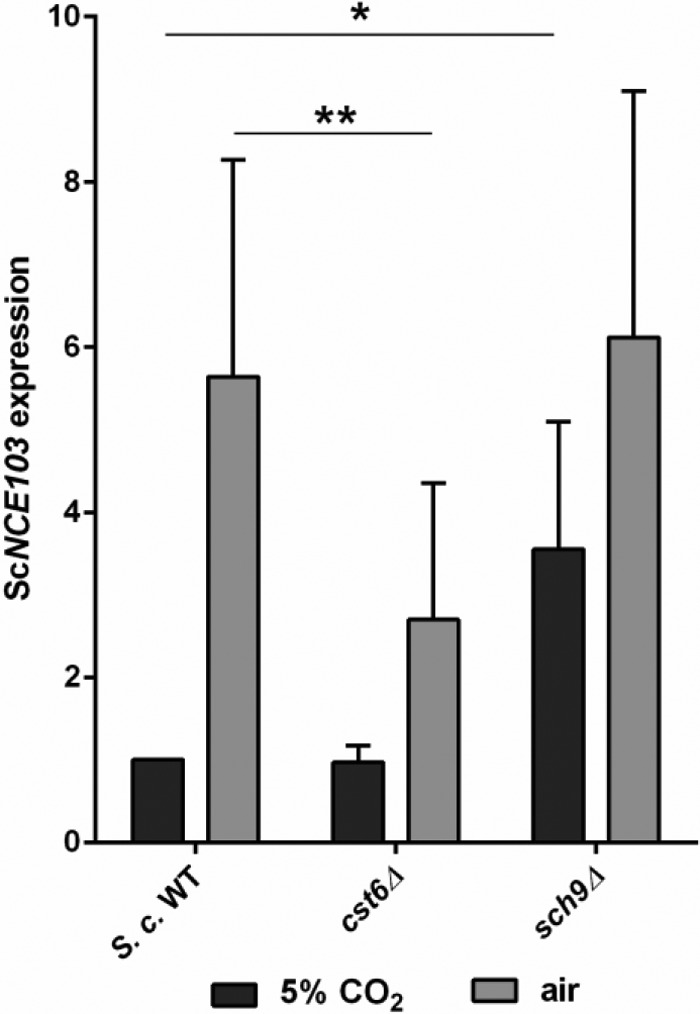
Mutant library screening reveals putative Cst6 kinase. The *S. cerevisiae* wild type (WT) and mutants were grown in 5% CO_2_ to the exponential phase and were either maintained at 5% CO_2_ or transferred to air for 60 min. Sc*NCE103* expression was measured by qRT-PCR and normalized to WT expression under 5% CO_2_ (for all data, see Table S2). ScSch9 was the most promising Cst6 kinase candidate. The bars show means ± standard deviations from ≥5 independent experiments. Significance against WT expression under the corresponding condition was calculated with a two-sided *t* test for unpaired samples and defined as *P* ≤ 0.05 (*) and *P* ≤ 0.01 (**).

Previous work suggested that phosphorylation of the Cst6/Rca1 family of transcription factors is key to fungal CO_2_ sensing, carbon fixation, and metabolism (17). To test this hypothesis, we opted for a high-throughput approach to identify candidate kinases/phosphatases. CO_2_ regulation in the model organism *S. cerevisiae* was previously reported to be particularly pronounced in response to CO_2_ variations of up to 20-fold between atmospheric and high CO_2_ (11). Using quantitative reverse transcription-PCR (qRT-PCR), we confirmed this finding and found that CA mRNA levels are induced more than 23-fold when cultures are transferred from 5% CO_2_ (*NCE103*^CO2^) to low-CO_2_ conditions (*NCE103*^air^), reaching maximum induction levels at 60 min (23.3 ± 4.9-fold induction) (see Fig. S1A in the supplemental material). Furthermore, we established that induction of *NCE103*^air^ was reduced in an *S. cerevisiae cst6*Δ mutant (Fig. 1) and that *CST6* expression itself was not affected by CO_2_ levels (Fig. S1B). Since CA expression by CO_2_ is conserved in these ascomycete yeasts, we decided to screen an *S. cerevisiae* kinase and phosphatase deletion mutant library of 155 strains (see Table S1 in the supplemental material) and quantify CO_2_-dependent gene regulation (see Table S2 in the supplemental material for all data). Deletion of a Cst6 kinase should result in derepression of CA in high CO_2_ and consequently increased *NCE103*^CO2^ expression at levels comparable to growth in air (see Fig. S2 in the supplemental material) (17). Accordingly, mutants with mean *NCE103*^CO2^ expression levels twice as high (≥2.0-fold) as the wild type (WT) were considered putative kinase candidates. Of the 155 strains screened, 5 mutants met these criteria: the *tpd3*Δ, *ptp1*Δ, *bud32*Δ, *sch9*Δ, and *ptk2*Δ strains (Fig. 1; see Table S2 and Table S3 in the supplemental material). Among those, the *sch9*Δ mutant showed the highest upregulation of Sc*NCE103*^CO2^ expression (3.55 ± 1.55-fold), whereas Sc*NCE103*^air^ expression (6.12 ± 2.98-fold) was similar to that of the WT. This deregulation pattern resulted in a low fold change between Sc*NCE103*^air^ and Sc*NCE103*^CO2^ of 1.91 ± 0.7-fold (Fig. 1; Table S2). Furthermore, the *sch9*Δ strain did not display general growth defects, and *SCH9* encodes a kinase known to be involved in stress response via nutritional sensing in *S. cerevisiae* and adaptation to hypoxia in *C. albicans* (20–24), making Sch9 the most probable candidate as Cst6 kinase. To confirm our hypothesis, we analyzed the Nce103 protein level and fluorescence levels in a CA promoter-green fluorescent protein (GFP) fusion, then carried out immunoprecipitation (IP) experiments, and finally showed that Sch9 phosphorylates Cst6.

10.1128/mBio.02211-16.1 FIG S1  CO_2_-dependent time course expression of Sc*NCE103* and Sc*CST6*. The *S. cerevisiae* wild type was grown in 5% CO_2_ to the exponential phase and was either maintained at 5% CO_2_ or transferred to air. Time course expression of Sc*NCE103* (A) and Sc*CST6* (B) was measured by qRT-PCR and normalized to expression at time point 0 in 5% CO_2_. Sc*NCE103* mRNA levels increase significantly in air; the highest expression was measured after 60 min. In contrast, Sc*CST6* expression is independent of CO_2_ levels. The graphs show means ± standard deviations. Significance against expression in 5% CO_2_ was defined as *P* ≤ 0.05 (*) and *P* ≤ 0.01 (**). *n* = 3. Download FIG S1, PDF file, 0.1 MB. Copyright © 2017 Pohlers et al. 2017 Pohlers et al. This content is distributed under the terms of the Creative Commons Attribution 4.0 International license.

10.1128/mBio.02211-16.2 TABLE S1  Strains, plasmids, and primers used in the study. Download TABLE S1, PDF file, 0.2 MB. Copyright © 2017 Pohlers et al. 2017 Pohlers et al. This content is distributed under the terms of the Creative Commons Attribution 4.0 International license.

10.1128/mBio.02211-16.3 TABLE S2  Mean Sc*NCE103* expression and standard deviation (SD) of *S. cerevisiae* kinase/phosphatase mutants in a changing CO_2_ environment. Download TABLE S2, PDF file, 0.2 MB. Copyright © 2017 Pohlers et al. 2017 Pohlers et al. This content is distributed under the terms of the Creative Commons Attribution 4.0 International license.

10.1128/mBio.02211-16.4 FIG S2  Workflow of mutant library screening for Cst6 kinase identification. *S. cerevisiae* wild-type and deletion mutant strains were grown in 5% CO_2_ to exponential phase and were either maintained at 5% CO_2_ or transferred to air for 60 min. RNA was extracted, and Sc*NCE103* expression was analyzed by qRT-PCR. Data were normalized to WT expression in 5% CO_2_. Putative Cst6 kinase deletion mutants were identified according to elevated Sc*NCE103* expression in 5% CO_2_ compared to the WT. Download FIG S2, PDF file, 0.04 MB. Copyright © 2017 Pohlers et al. 2017 Pohlers et al. This content is distributed under the terms of the Creative Commons Attribution 4.0 International license.

10.1128/mBio.02211-16.5 TABLE S3  Kinase candidate genes identified in mutant library screening. Download TABLE S3, PDF file, 0.1 MB. Copyright © 2017 Pohlers et al. 2017 Pohlers et al. This content is distributed under the terms of the Creative Commons Attribution 4.0 International license.

### Loss of Sch9 deregulates CO_2_ sensing.

**FIG 2:**
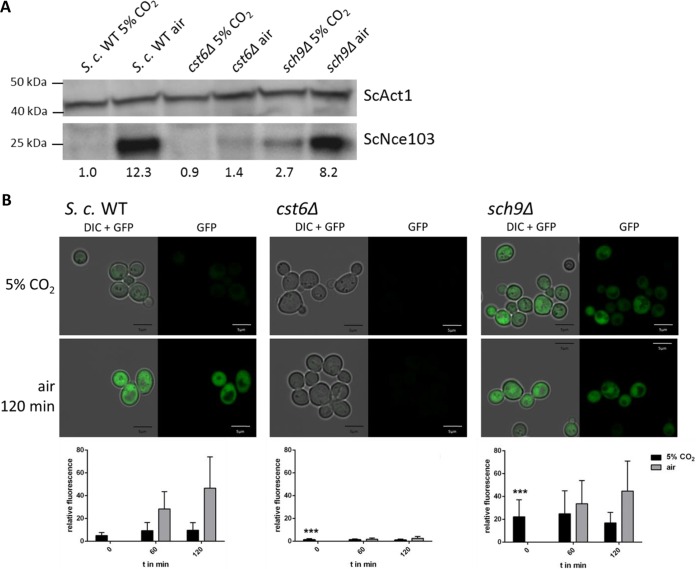
Loss of ScSch9 enhances ScNce103 protein level and Sc*NCE103* promoter activation under 5% CO_2_. (A) Endogenous ScNce103 levels of the WT and mutants cultivated under 5% CO_2_ and air conditions were detected by Western blot analysis. The *sch9*Δ mutant showed elevated ScNce103 in 5% CO_2_ compared to the WT. Nce103 levels relative to the loading control and normalized to WT in 5% CO_2_ are indicated below the blot. (B) The *S. cerevisiae* WT and mutants were transformed with GFP under the control of the Sc*NCE103* promoter and grown under 5% CO_2_. Cells were either maintained under this condition or transferred to air for up to 120 min. Fluorescence was detected at the time point of the switch (0 min) and after 60 min and 120 min. Quantification was done by measuring the fluorescence intensity of at least 50 cells per time point. DIC, differential inference contrast. The significance of mutant against WT fluorescence at time point 0, calculated by two-sided *t* test for unpaired samples, was defined as *P* ≤ 0.001 (***).

Using a specific antibody against *S. cerevisiae* CA, we measured protein levels in the *sch9*Δ mutant cultivated under different CO_2_ conditions. In contrast to the WT, Nce103 levels in the *sch9*Δ strain were elevated regardless of the strain being exposed to low or high CO_2_ (Fig. 2A). Similar to *NCE103* gene expression, the Nce103 protein level of the *sch9*Δ strain was not strongly affected by environmental CO_2_. Next we transformed WT, *cst6*Δ and *sch9*Δ cells with a plasmid containing GFP under control of the Sc*NCE103* promoter and exposed to low and high concentrations of CO_2_. GFP expression was analyzed using confocal microscopy (Fig. 2B). GFP fluorescence of WT cells under 5% CO_2_ was constantly very low, but fluorescence increased after incubation in air (6-fold after 60 min and 9-fold after 120 min) (Fig. 2B). The persistent increase over time is likely due to the stability of GFP. In contrast, the *cst6*Δ strain showed nearly undetectable fluorescence levels under both conditions. The *sch9*Δ strain exhibited significantly higher fluorescence in 5% CO_2_ compared to the WT, but there were also higher differences in intensity between cells. Fluorescence in air was comparable to that of the WT. Noticeably, whereas *NCE103* expression levels of the *cst6*Δ mutant in air and the *sch9*Δ mutant in 5% CO_2_ were not significantly different (Fig. 1), the GFP reporter signal for the *cst6*Δ mutant in air was lower than that for the *sch9*Δ mutant in high CO_2_. This could be due to the intrinsically high variation of *NCE103* expression in air, which ranged from 3-fold to 10-fold when normalized to expression in 5% CO_2_. Furthermore, the experimental settings differ, with the GFP reporter being expressed from a plasmid rather than from its native locus. However, results for *NCE103* levels in 5% CO_2_ are consistent in both assays and confirm that loss of Sch9 leads to a loss of CO_2_-dependent *NCE103* repression.

### Sch9 is the Cst6 kinase.

**FIG 3:**
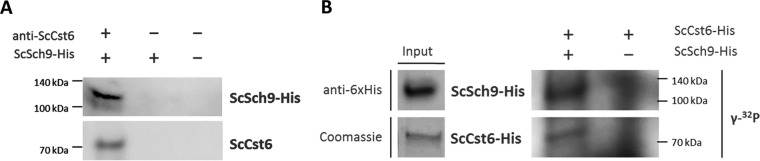
ScSch9 interacts with and phosphorylates ScCst6. (A) ScCst6 was immunopurified from *S. cerevisiae* using specific antibodies and subsequently incubated with purified ScSch9-His. ScSch9 was only detectable when ScCst6 was previously bound. (B) For *in vitro* phosphorylation assays, ScSch9-His was immunopurified from *S. cerevisiae* grown under 5% CO_2_ and incubated with recombinant ScCst6-His in the presence of [γ-^32^P]ATP. Incorporation of γ-^32^P was detected by autoradiography. A clear band for radioactively labeled ScCst6 was visible, indicating phosphorylation of ScCst6 by ScSch9.

To demonstrate physical interaction between Cst6 and Sch9, immunoprecipitation experiments were performed. Endogenous ScCst6 was precipitated from WT lysate using a specific anti-Cst6 antibody. Immunocomplexes were bound to Sepharose beads and incubated with recombinantly expressed Sch9-His. After extensive washing, Western blot analysis of bound proteins was performed (Fig. 3A). Beads without addition of anti-Cst6 antibody did not show a signal for either Cst6 or Sch9. Addition of Sch9-His without prior binding of Cst6 by antibodies also did not exhibit any signal, which excluded the possibility of unspecific binding. Importantly, when Cst6 was previously bound by anti-Cst6 antibody, we could detect specific binding of Sch9, thus demonstrating the ability of Sch9 to bind its expected substrate, ScCst6.

To provide further evidence that Sch9 is the Cst6 kinase, we carried out phosphorylation experiments. We overexpressed Sch9-His in *S. cerevisiae* under control of the inducible Sc*GAL1* promoter. Sch9 expression was induced by shifting the cells from glucose- to galactose-containing medium. *S. cerevisiae*-overexpressing cells were cultivated in 5% CO_2_, assuming that the kinase should be most active under this condition. ScSch9-His was purified from cell lysate via immunoprecipitation. Successful purification was followed by Western blot analysis resulting in a strong single band at ~120 kDa in the bead-bound fraction (Fig. 3B, left panel). Immunocomplexes with bound kinase were used for the radioactive kinase assay. The substrate Cst6-His was recombinantly expressed and purified using affinity chromatography. Purity was verified by Coomassie staining, represented by a single band at ~70 kDa (Fig. 3B, left panel). Sch9-His and Cst6-His were incubated in the presence of [γ-^32^P]ATP before separation by 4 to 20% SDS-PAGE. By means of autoradiography, incorporation of γ-^32^P was visualized. In control experiments, where the putative kinase ScSch9 was absent, no definite bands were visible, but a high background corresponding to nonincorporated radioactivity was seen (Fig. 3B, right panel). If Sch9 was present, two distinct bands of ~70 kDa and ~120 kDa were apparent, representing Cst6 and Sch9, respectively. These data demonstrate the phosphorylation of Cst6 by Sch9 *in vitro* and, furthermore, imply an extensive autophosphorylation of ScSch9, which was previously reported by Huber et al. (25).

### Cst6 phosphorylation analysis demonstrates that CO_2_ signals via phosphorylation of S266.

**FIG 4:**
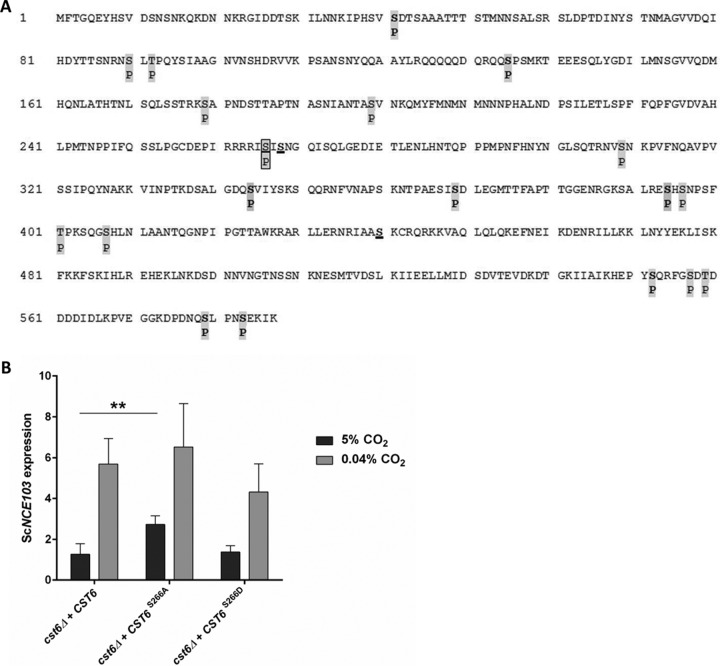
ScCst6 phosphorylation at position S266 is crucial for Sc*NCE103* regulation. (A) ScCst6-His, overexpressed in the WT under 5% CO_2_, was affinity purified and subjected to SDS-PAGE. Corresponding bands were excised and digested with trypsin-LysC, AspN, GluC, or LysargiNase. Additionally, in-solution digestion for trypsin-LysC and GluC was performed. Phosphorylated peptides were enriched and analyzed by LC-MS/MS. Nineteen different phosphorylation sites, indicated by “P,” were identified in at least 2 of 10 independent experiments. Newly identified phosphorylation sites are shown in boldface, and previously known sites are not in boldface. Underlined are the 3 serine residues that were conserved in *S. cerevisiae*, *C. glabrata*, and *C. albicans*. Among them, only S266 was found to be phosphorylated in the WT, noted within a box. (B) Site-specific mutation of S266 was performed to investigate direct influence on Sc*NCE103* expression under changing CO_2_ conditions. Expression levels were normalized to WT Sc*NCE103* expression in 5% CO_2_. Changing S266 to alanine (*cst6Δ + CST6*^S266A^) showed a significant upregulation of Sc*NCE103*^CO2^, while Sc*NCE103*^air^ levels were unaltered. This is comparable to expression changes of the *sch9*Δ mutant and proves the critical role of Cst6 S266 for CA regulation. The bars show means ± standard deviations from 5 independent experiments. Significance against the *cst6Δ + CST6* strain, calculated by a two-sided *t* test for unpaired samples, was defined as *P* ≤ 0.01 (**).

CO_2_ regulation of fungal CA expression is conserved, and *S. cerevisiae* Cst6 is an orthologue to Rca1 in *C. albicans* and *C. glabrata*. Accordingly we hypothesized fungal CO_2_-sensing signals via phosphorylation of defined Cst6/Rca1 amino acid residues. Comparison of the Cst6/Rca1 protein sequences identified three conserved serine residues at positions S124, S126, and S222 in *C. albicans* Rca1 corresponding to S266, S268, and S440 in *S. cerevisiae* Cst6. Previous work suggested that S124 but not S126 or S222 disrupts CA regulation in *C. albicans* (17). Hence, we assumed that phosphorylation of S124 regulates fungal CO_2_ sensing. In order to analyze the phosphorylation probability of the three serine residues, we overexpressed Cst6-His in the *S. cerevisiae* WT in 5% CO_2_, assuming that phosphorylation status is at maximum. Cst6-His was affinity purified via immobilized metal ion affinity chromatography (IMAC) and subjected to 4 to 20% SDS-PAGE. Total protein was Coomassie stained, corresponding bands were excised, and trypsin-LysC, AspN, LysargiNase, or GluC digestion was performed to achieve high sequence coverage. In addition, an in-solution digestion with trypsin-LysC and GluC was done to also detect peptides of extended length. Phosphopeptides were enriched using TiO_2_ and analyzed by liquid chromatography-tandem mass spectrometry (LC-MS/MS). Our results clearly showed that multiple sites of the Cst6 protein can be subject of phosphorylation. In the WT, we detected 19 different phosphorylation sites in at least two independent experiments (Fig. 4A). Thereof, 11 residues were previously identified by high-throughput approaches (26, 27). We identified another 8 sites, which were unknown to be phosphorylated, printed in boldface in Fig. 4A. In Cst6, the conserved serine residues correspond to S266, S268, and S440. Of those, only serine at position 266 was found to be phosphorylated in *S. cerevisiae* in 5% CO_2_, while S268 and S440 were found to be unphosphorylated. In addition, the number of detected phosphorylations suggests the possibility of additional kinases being involved in regulation of Cst6, although we cannot draw any conclusions about biological relevance from the phosphorylation site mapping.

S266 is the only residue within Sc*CST6* matching the Sch9 consensus motif R-(R/K)-X-S (28). Hence, we mutated serine at position 266 to alanine (S266A) or aspartic acid (S266D) and cloned these sequences or WT *CST6* into the *cst6*Δ mutant to prove influence on Sc*NCE103* expression. The *cst6*Δ mutant reverted with WT *CST6* showed Sc*NCE103* expression comparable to that of the WT (Fig. 4B). Phosphoablative mutation of S266 to A led to a significant upregulation of Sc*NCE103*^CO2^ (2.73 ± 0.43-fold), while Sc*NCE103*^air^ (6.52 ± 2.12-fold) expression was unaltered. Interestingly, the pattern of expression of the *cst6Δ + CST6*^S266A^ strain corresponds to the expression pattern of the *sch9*Δ mutant. This confirms our hypothesis and emphasizes S266 to be phosphorylated in 5% CO_2_ in order to inhibit *NCE103* expression. Phosphomimetic mutation of *CST6* (*CST6*^S266D^) led to slightly decreased Sc*NCE103*^air^ expression in comparison to WT *CST6*, but differences were not statistically significant. This might point to alternative regulation mechanisms regulating *NCE103* expression, especially under the air condition.

### CO_2_ sensing is conserved in yeast.

**FIG 5:**
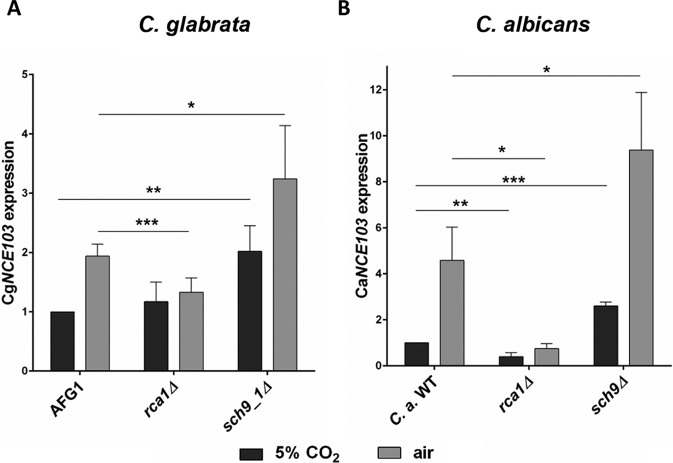
*SCH9* function in CO_2_ signaling is conserved in pathogenic yeasts. *C. glabrata* (A) and *C. albicans* (B) strains were grown under different CO_2_ conditions, and expression of *NCE103* was measured by qRT-PCR. Cg*NCE103* expression was normalized to control expression (AFG1), and Ca*NCE103* expression was normalized to the *C. albicans* WT, cultivated in 5% CO_2_. Both species showed comparable regulation mechanisms. Deletion of *RCA1* led to significantly decreased *NCE103*^air^ expression. *sch9*Δ mutants of *C. glabrata* and *C. albicans* showed increased *NCE103*^CO2^ expression, consistent with findings for *S. cerevisiae*. This suggests a conserved role of *SCH9* in CO_2_ signaling in all three yeast species. The bars show means ± standard deviations from ≥4 independent experiments. Significance against control expression, calculated by two-sided *t* test for unpaired samples, was defined as *P* ≤ 0.05 (*), *P* ≤ 0.01 (**), and *P* ≤ 0.001 (***).

Our findings and previous work suggest that CO_2_-dependent regulation mediated via Cst6/Rca1 is conserved in some yeast species (17, 18). Due to the closer phylogenetic relationship between *S. cerevisiae* and *C. glabrata*, we first analyzed the influence of *C. glabrata SCH9* (Cg*SCH9*) on Cg*NCE103* expression under changing CO_2_ conditions. Expression of Cg*NCE103* in 5% CO_2_ and air after 60 min was quantified by qRT-PCR according to the previously described protocol for *S. cerevisiae* (Fig. 5A). The *C. glabrata* control strain, AFG1, revealed a 2-fold increase of Cg*NCE103*^air^ compared to Cg*NCE103*^CO2^ expression. This increase was completely absent in the *rca1*Δ mutant. In fact, Cg*NCE103* expression of the *rca1*Δ mutant in air was as low as in 5% CO_2_. This is in contrast to the *S. cerevisiae cst6*Δ regulation pattern, where at least a small upregulation in air was observed. As expected, deletion of Cg*Sch9* led to significantly elevated Cg*NCE103*^CO2^ levels (2.02 ± 0.43-fold) compared to the control. Noteworthy and in contrast to the pattern found in* S. cerevisiae*, Cg*NCE103*^air^ expression was also increased. However, despite these differences, Sch9 clearly affects *NCE103* regulation in *C. glabrata*.

We also verified the influence of *SCH9* on *C. albicans NCE103* (Ca*NCE103*) using similar protocols (Fig. 5B). The *C. albicans* WT exhibited a 4.6-fold increase of Ca*NCE103* expression in cells transferred to air conditions. In comparison, deletion of Ca*RCA1* resulted in significantly lower Ca*NCE103*^air^ expression. A *C. albicans sch9*Δ mutant strain (24) showed significantly increased Ca*NCE103*^CO2^ expression (2.61 ± 0.16-fold), similar to the effects of *SCH9* deletion in *S. cerevisiae* and *C. glabrata*. Remarkably, also Ca*NCE103*^air^ levels were elevated, which coincides with what we found for *C. glabrata*, but not for *S. cerevisiae*. Taken together, these findings clearly indicate a conserved mechanism of CO_2_ adaptation in pathogenic and nonpathogenic yeast species and emphasize Sch9 is key to CO_2_ sensing in all three species.

### Lipid signaling via Pkh1/2 but not TORC1 regulates fungal CO_2_ sensing.

Previous work showed that in *S. cerevisiae* Sch9 is regulated by target-of-rapamycin (US approved name [USAN] sirolimus)-complex 1 (TORC1) and homologues of the mammalian 3-phosphoinositide-dependent protein kinase (mPDK1), Pkh1 and Pkh2, which phosphorylate Sch9 at specific sites (29, 30). TORC1 phosphorylates at least 6 residues of Sch9 (S711, T723, S726, T737, S758, and S765) (31), while ScPkh1/2 phosphorylates Sch9 at the *PDK1* site T570 (32).

**FIG 6:**
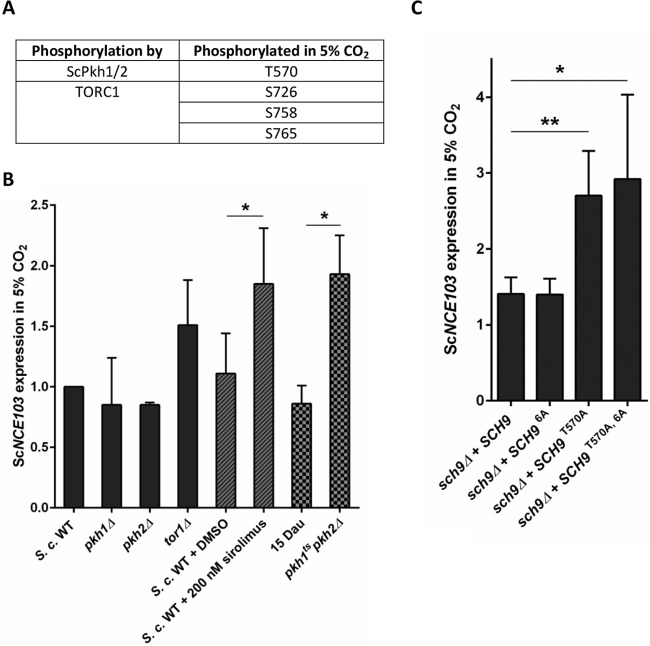
Pkh1/2 but not TORC1 mediates activation of ScSch9-ScCst6 signaling in 5% CO_2_. (A) Phosphorylation of ScSch9 in 5% CO_2_ was detected using LC-MS/MS. Phosphorylation of residues known to be addressed by ScPkh1/2 and TORC1 was detected. (B) Sc*NCE103*^CO2^ expression of the WT and ScSch9 activator mutants was measured by qRT-PCR and normalized to WT expression. Sc*TOR1*, Sc*PKH1*, or Sc*PKH2* single mutants did not change Sc*NCE103*^CO2^ expression significantly. Furthermore, *S. cerevisiae* WT was treated with 200 nM sirolimus to inactivate TORC1 or DMSO as a solvent control. Sirolimus treatment led to upregulation of Sc*NCE103*^CO2^ expression, but it does not reach the Sc*NCE103*^CO2^ levels of the *sch9*Δ mutant. Because of Sc*PKH1/2* redundancy, a temperature-sensitive double mutant in the background of 15 Dau was tested and revealed significantly increased Sc*NCE103*^CO2^ levels. The bars show means ± standard deviations from ≥3 independent experiments. Significance against expression of the WT plus DMSO or 15 Dau, calculated by two-sided *t* test for unpaired samples, was defined as *P* ≤ 0.05 (*). (C) Sc*NCE103* expression of the *sch9Δ + SCH9* strain and revertants with* SCH9* possessing S/T-to-A mutations of known activation sites was measured in changing CO_2_ and normalized to WT expression. Integration of* SCH9* in the *sch9*Δ strain restored the WT regulation pattern of Sc*NCE103*. Phosphoablative mutation of the 6 phosphorylation sites known to be addressed by TORC1 (S711A, T723A, S726A, T737A, S758A, and S765A), indicated as *SCH9*^6A^, showed no expression changes. Mutation of the *PKH1/2* site T570 increased Sc*NCE103*^CO2^ expression significantly, indicating reduced activation of ScSch9. Combination of mutations of phosphorylation sites addressed by both activators, *SCH9*^T570A, 6A^, had no additional effect on Sc*NCE103*^CO2^ expression, but Sc*NCE103*^air^ expression was significantly enhanced. The bars show means ± standard deviations from ≥4 independent experiments. Significance against the *sch9Δ + SCH9* strain, calculated by two-sided *t* test for unpaired samples, was defined as *P* ≤ 0.05 (*) and *P* ≤ 0.01 (**).

Analysis of LC-MS/MS data of overexpressed Sc*CST6*-His allowed us to detect phosphorylated peptides specific for endogenous ScSch9. This enabled analysis of Sch9 phosphorylation *in vivo* to draw conclusions about putative activators. We detected the PDK1 site T570 and 3 of the phosphorylation sites addressed by TORC1 (S726, S758, and S765) to be phosphorylated in 5% CO_2_ (Fig. 6A). Therefore, we hypothesized that TOR and lipid signaling is involved in fungal CO_2_ sensing and that loss or inhibition of Sch9 upstream regulators mimics the *sch9*Δ mutation. Because activation of Sch9 is especially important under 5% CO_2_, we concentrated on changes of *NCE103*^CO2^ expression (see Table S2 and Table S4 in the supplemental material for *NCE103*^air^ expression). Notably, the *tor1*Δ mutant had been included in our initial qRT-PCR screening and showed slightly but not significantly increased CA expression in high CO_2_ (Fig. 6B). Catalytic subunits of TORC1 are either ScTor1 or ScTor2, and TORC1 is inhibited by sirolimus (33). Since Tor2 is an essential gene in *S. cerevisiae*, we investigated the impact of TORC1 signaling on fungal CO_2_ sensing via Sch9 by using sirolimus. Inhibition of TORC1 by sirolimus aggravated the effect of *tor1*Δ on CA gene expression and led to a significant upregulation of *NCE103*^CO2^ (1.85 ± 0.46-fold) (Fig. 6B). However, it did clearly not reach the* NCE103*^CO2^ levels of the *sch9*Δ mutant. *PKH1*/*PKH2* single mutants did not show altered *NCE103*^CO2^ expression (Fig. 6B), probably because of Pkh1/2 redundancy in *S. cerevisiae* (34). As a conventional Pkh1/2 double deletion mutant is known to be lethal in *S. cerevisiae* (35), we used a temperature-sensitive double mutant (35) to investigate the influence of Pkh1/2-mediated activation of Sch9 on *NCE103*^CO2^ expression. Indeed, expression levels of *NCE103*^CO2^ increased significantly to 2-fold in the *pkh1*^*ts*^* pkh2*Δ mutant compared to its control strain, 15 Dau. This implies that Sch9-mediated effects on CA expression could be dependent on both Sch9 regulators TORC1 and Pkh1/2.

10.1128/mBio.02211-16.6 TABLE S4  Mean Sc*NCE103* expression and standard deviation (SD) of *S. cerevisiae SCH9* regulator mutants and *S. cerevisiae* under rapamycin (USAN sirolimus) treatment. Download TABLE S4, PDF file, 0.1 MB. Copyright © 2017 Pohlers et al. 2017 Pohlers et al. This content is distributed under the terms of the Creative Commons Attribution 4.0 International license.

To analyze the influence of Sch9 activation by TORC1 and Pkh1/2 more precisely, we investigated *NCE103* expression of strains with site-specific mutations of known Sch9 phosphorylation sites addressed by TORC1 and Pkh1/2. As a control, the *sch9*Δ mutant reverted with WT *SCH9* showed *NCE103* expression levels in 5% CO_2_ and air comparable to those of the WT strain. Surprisingly, also phosphoablative mutation of all 6 TORC1 sites (S711A, T723A, S726A, T737A, S758A, and S765A), referred to as *SCH9*^6A^, resulted in WT-like *NCE103* expression levels (Fig. 6C). In contrast, T570A mutation of the PDK1 site resulted in significantly elevated Sc*NCE103*^CO2^ expression (2.7 ± 0.59), while Sc*NCE103*^air^ expression was not changed. This is comparable with the deregulation pattern of the *sch9*Δ mutant. Additional S/T-to-A mutation of the 6 TORC1 sites within *SCH9* (*SCH9*^T570A, 6A^) showed no additive effect on Sc*NCE103* expression (Fig. 6C).

Taken all together, we concluded that phosphorylation of Sch9 by Pkh1/2 at T570 is crucial for Sch9 activation in a 5% CO_2_ environment in order to decrease Sc*NCE103* expression. Although phosphorylation sites addressed by TORC1 were detected in Sch9, CO_2_-dependent activation of Sch9 and thus CO_2_ adaptation seems to be independent of TORC1.

## DISCUSSION

Sensing and adapting to alterations in ambient CO_2_ are of paramount importance for all living organisms. Fungi adjust their metabolism to CO_2_ availability, and CAs are essential in this process. CAs catalyze the reversible conversion of mobile CO_2_ to HCO_3_^−^, and disruption of their function using CA inhibitors or deletion distorts fungal metabolism or abolishes growth altogether.

Expression of the fungal CA *NCE103* is regulated by the transcription factors Cst6 and Rca1, which belong to the ATF/CREB family (17). To identify enzymes responsible for Cst6/Rca1 phosphorylation and unravel fungal CO_2_ sensing, we screened a kinase/phosphatase mutant library in the model organism *S. cerevisiae*. The library consisted of 111 kinase and 44 phosphatase mutants, and Sch9 was the most promising identified kinase candidate.

Sch9 regulates ribosome biogenesis, cell cycle progression, and life span and is involved in heat shock, osmotic and oxidative stress response by integrating nutrient signals (20–23, 36–38). Several lines of evidence support our hypothesis that Sch9 is indeed the Cst6 kinase and relays fungal CO_2_ sensing: immunoprecipitation using endogenous Cst6 showed a physical interaction between Cst6 and Sch9. Furthermore, *in vitro* phosphorylation showed that Sch9 mediates the incorporation of radioactively labeled phosphate groups into Cst6, clearly demonstrating the kinase activity. Finally, the preferred consensus motif for Sch9 phosphorylation events is R-(R/K)-X-S (28).

Our work provides evidence that regulation of *NCE103* in *S. cerevisiae*, *C. glabrata*, and *C. albicans* is conserved. Deletion of Sc*SCH9*, Cg*SCH9*, and Ca*SCH9* affected *NCE103* expression in comparable ways as they lead to a significant upregulation of *NCE103*^CO2^ (Fig. 1 and 5). Previous work provided some indirect evidence suggesting that S124 in *C. albicans* may be subject to phosphorylation impacting Rca1/Cst6 function (17). S124 is conserved in *S. cerevisiae* Cst6 (corresponding position S266) and *C. glabrata* Rca1 (S387). We now show proof that S266, but not other candidate residues, is phosphorylated in *CST6* under elevated CO_2_ and thus provide a molecular explanation for the conservation of CO_2_ signaling in yeast (Fig. 4). Furthermore, we showed that phosphorylation of Sc*CST6* at S266 is crucial for repression of *NCE103*^CO2^, because the phosphoablative mutation S266A resulted in increased *NCE103*^CO2^ expression. This is in accordance with the results Cottier et al. found for S124 in *C. albicans* Rca1 (17).

**FIG 7:**
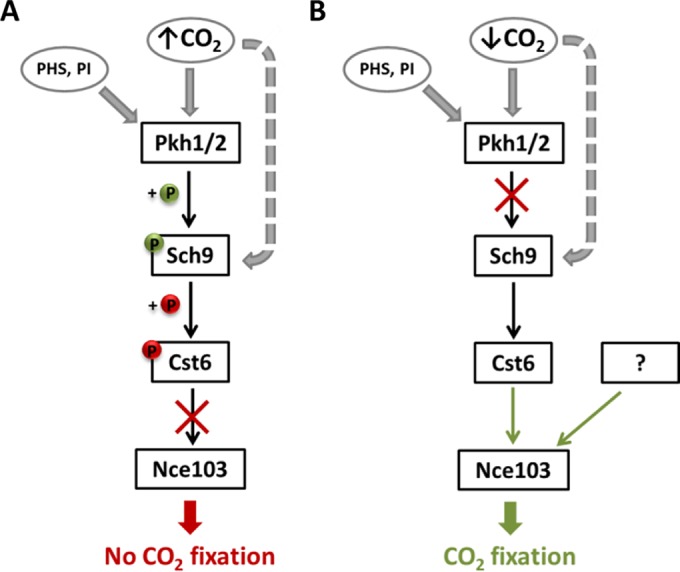
Model of Nce103 regulation under changing CO_2_ conditions. (A) In the 5% CO_2_ environment, Sch9 is activated by phosphorylation of T570 via Pkh1/2, whereby Pkh1/2 activity is influenced by phytosphingosine (PHS) and phosphatidylinositol (PI). Moreover, a direct influence of CO_2_ levels on Sch9 activation is possible. Activated Sch9 phosphorylates the transcription factor Cst6, which is subsequently not able to induce *NCE103* expression. This results in very low Nce103 levels, which do not enhance CO_2_ fixation. (B) Under air conditions, Sch9 is not activated by Pkh1/2 and does not inhibit Cst6. Active Cst6 induces *NCE103* expression and enhances the Nce103 protein level, leading to fixation of CO_2_. Furthermore, Nce103 seems to be induced by another, so far unknown mechanism.

Sch9 is a member of the AGC family (homologous to protein kinases A, G, and C) of proteins. It is conserved in *S. cerevisiae*, *C. glabrata*, and *C. albicans* but is also found in less-related fungal species like *Fusarium graminearum* (39). In mammalian cells, it has been hypothesized that PKB/Akt is the Sch9 homologue (40), although later studies suggest that Sch9 is more closely related to S6K1 (31), which acts downstream of TORC1. The best-characterized regulator of CA in human cells is HIF-1α, which induces human CA IX expression in response to hypoxia (41, 42). HIF-1α has been found to be regulated by mTOR, which suggested the assumption that Cst6/Rca1 regulation in yeast is also TOR dependent. Surprisingly, our experiments demonstrated that Sch9 phosphorylation by TORC1 does not contribute to CO_2_-dependent *NCE103* regulation (Fig. 6C). Indeed, inhibition of TORC1 by sirolimus showed effects on *NCE103*^CO2^ (Fig. 6B), but these effects seem not to be dependent on Sch9 signaling. Furthermore, it was shown that addition of sirolimus is not always similar to inhibition of TORC1 (43). Instead of TORC1, phosphorylation by Pkh1/2 at T570 seems to be the driving force for Sch9 activation in order to repress *NCE103* expression under 5% CO_2_ (Fig. 7). It should be considered that Pkh1/2 might not be the sole activator of Sch9. Deletion of *SCH9* results in a 3.55-fold upregulation of Sc*NCE103*^CO2^, whereas increase following deletion of *PKH1/2* was only 1.93-fold. Also T570A mutation of Sch9 leading to unresponsiveness to Pkh1/2 activation did not reach the high *NCE103*^CO2^ levels. Thus, there is the possibility that Sch9 is directly influenced by CO_2_ levels and not only via Pkh1/2 (Fig. 7). An influence of oxygen levels on Sch9 activity should also be considered: it has been previously reported that Sch9 inhibits hyphae formation in the fungal pathogen *C. albicans* under high-CO_2_ conditions (>1%) and hypoxia (<10%) but not under either condition alone (24).

It is noteworthy that another candidate identified in our screen was ScTpd3, which is a part of the protein phosphatase 2A complex. Although no kinase function has been reported, the protein phosphatase 2A complex is linked to nutrient signaling via Tor (44) and is suspected to dephosphorylate Sch9. Overexpression of Sc*TPD3* and deletion of *SCH9* result in comparable phenotypes (45). Thus, ScTpd3 might indeed play a role in *NCE103* regulation—maybe as a regulator of ScSch9.

The complexity of *NCE103* regulation is further confirmed by our finding that despite the absence of ScCst6 as a transcriptional activator, we could observe upregulation of Sc*NCE103*^air^ resulting in elevated ScNce103p, although this was clearly less pronounced than in the WT (Fig. 2A). Therefore, besides the ScSch9-ScCst6 pathway, there seem to be other mechanisms regulating Sc*NCE103*, maybe another—so far unknown—transcription factor (Fig. 7B). This could be an explanation for the viability of *cst6*Δ mutants even under air conditions, whereas *nce103*Δ mutants are only able to grow in elevated CO_2_ environment (16). Only a moderate induction of *NCE103* expression seems to be sufficient for fungal survival, but it will result in growth delay and, probably, affect other cell properties.

In this study, we provide insight into fungal CO_2_ sensing and show that lipid signaling activating Pkh1/2 regulates metabolic adaptation via Sch9. More work is needed to identify the precise molecular events and complex feedback mechanisms involved in CO_2_ signal reception and transduction to Sch9. However, the Sch9-Cst6/Rca1 cascade is conserved in yeasts and central to fungal metabolism, and Rca1 has previously been reported to play a role in biofilm formation, which is instrumental in fungal pathogenesis (46). Thus, targeting fungus-specific components in organisms CO_2_ sensing, such as CA or Rca1/Cst6, constitutes a valid approach to new treatment option.

## MATERIALS AND METHODS

### Strains, plasmids, and primers.

For the strains, plasmids, and primers used in this study, see Table S1.

### Sc*NCE103* expression analysis.

Exponential cultures of *S. cerevisiae* WT (BY4741) were cultivated at 37°C as duplicates in 5% CO_2_ and transferred to air for 10, 30, 60, 90, 120, or 180 min to induce Sc*NCE103* expression. Pellets were harvested, and RNA was extracted using a hot phenol-chloroform method (47). The quantity of RNA was measured with NanoDrop 2000 (Thermo Fisher Scientific, Waltham, MA), and 100 ng/µl RNA was utilized as the template for qRT-PCR using a Brilliant III Ultra Fast SYBR green qRT-PCR kit on a Stratagene Mx3005P (both Agilent Technologies, Santa Clara, CA). Gene expression was calculated by the ΔΔ*C*_*T*_ method (48) using Sc*ACT1* as the housekeeping gene. The same procedure was done for the screening of *S. cerevisiae* kinase/phosphatase mutant library (Euroscarf, Frankfurt, Germany). Exponential cultures were transferred to air for 60 min to induce the highest Sc*NCE103* expression. Sc*NCE103* expression of mutants was normalized to WT expression in 5% CO_2_ (Fig. S2). The mutants were tested at least twice, those that showed dysregulation were run 3 to 6 times. Expression of the WT strain was measured in every qRT-PCR as an internal control. For sirolimus experiments, 200 nM sirolimus of a 1 mM stock solution in dimethyl sulfoxide (DMSO) was added. DMSO served as a solvent control. Sc*NCE103* expression of the 15 Dau and *pkh1*^*ts*^* pkh2*Δ strains was measured with slight differences in the protocol due to temperature sensitivity of the *pkh1*^*ts*^* pkh2*Δ mutant. Strains were preincubated overnight in 30°C and 5% CO_2_ and transferred to 37°C for 3 h to inhibit Pkh1 synthesis. Then samples were transferred to air for another hour to induce Sc*NCE103*.

### Expression and purification of His-tagged proteins from *E. coli*.

*S. cerevisiae CST6*, *NCE103*, and *SCH9* were synthesized with a C-terminal 6×His tag (GeneArt Gene Synthesis, Thermo Fisher Scientific) and cloned into *E. coli* expression vector p41. *NCE103* and *SCH9* were expressed in *E. coli* strain BL21(DE3) (New England Biolabs, Ipswich, MA). Bacteria were grown in LB medium (10 g/liter NaCl, 5 g/liter yeast extract, 10 g/liter tryptone [pH 7]) with 50 µg/ml ampicillin at 37°C. Sc*NCE103* expression was induced with 1 mM IPTG (isopropyl-β-d-thiogalactopyranoside) at an optical density at 600 nm (OD_600_) of 0.2 and cultivated overnight until harvesting. Expression of Sc*SCH9* was induced at an OD_600_ of 0.8 for 4 h at room temperature. Sc*CST6* expression was performed using* E. coli* Rosetta. Cells were cultivated in LB medium with 50 µg/ml ampicillin and 34 µg/ml chloramphenicol at 37°C until they reached an OD_600_ of 0.4. Expression was induced with 1 mM IPTG for 4 h at 37°C. Bacteria were harvested, resuspended in purification buffer (25 mM sodium phosphate [pH 7.4], 250 mM NaCl, 10 mM imidazole, 1 mM phenylmethylsulfonyl fluoride [PMSF]), and disrupted by freezing and thawing for a total of 3 rounds. Recombinant proteins were purified by IMAC using Ni-nitrilotriacetic acid (NTA) Sepharose (Biozol, Eching, Germany). His-tagged proteins were eluted with purification buffer containing 250 mM imidazole. For further purification of ScCst6-His, size exclusion chromatography using an Äkta with a Sephadex 200 GL column (both GE Healthcare, Little Chalfont, United Kingdom) was performed. Protein purity was determined by silver staining, and quantity was measured with a bicinchoninic acid (BCA) assay. At least 1 mg purified protein was used to produce specific rabbit antibodies against ScCst6-His and ScNce103-His (Thermo Fisher Scientific).

### Western blot analysis.

For detection of ScNce103 levels, 10 ml of *S. cerevisiae* exponential cultures was cultivated in 5% CO_2_ or transferred to air for 90 min. Cells were harvested, resuspended in 500 µl yeast lysis buffer (50 mM Tris-HCl, 150 mM NaCl, 0.1% Triton X-100, 1 mM dithiothreitol [DTT], 10% glycerol, PhosStop, Complete EDTA-free protease inhibitor, PMSF), and lysed with glass beads (diameter, 425 to 600 µm) five times for 1 min each with incubation on ice between lyses. Lysates were centrifuged (16,000 × *g*, 10 min, 4°C), and total protein was measured using a BCA protein assay kit (Thermo Fisher Scientific). After addition of 5× SDS loading dye and heating at 95°C for 5 min, samples (1 mg total protein) were subjected to 4 to 20% SDS-PAGE. Transfer to polyvinylidene difluoride (PVDF) membrane was performed using the Bio-Rad Wet/Tank blotting system for 1 h. Three percent nonfat dry milk in Tris-buffered saline (TBS) was used for membrane blocking and antibody binding. Membranes were incubated with custom-made rabbit anti-ScNce103 (1:1,000 [Thermo Fisher Scientific]) and mouse anti-β-actin (AB8224; 1:1,000 [Abcam, Inc., Cambridge, United Kingdom]) overnight at 4°C with gentle rotation. After incubation with fluorophore-labeled secondary antibodies (Jackson ImmunoResearch, West Grove, PA) for 2 h at 4°C, signals were detected with the FluorChem Q Imager (Alpha Innotec, Kasendorf, Germany). Quantitative analysis was done using the AlphaView software (ProteinSimple).

### *NCE103* promoter-driven GFP expression.

The *S. cerevisiae* WT, *cst6*Δ, and *sch9*Δ strains were transformed with pRS316 plasmid, containing GFP under control of the Sc*NCE103* promoter using the lithium-acetate (LiAc) method according to Gietz et al. (49). In brief, yeast cells of the early lag phase were harvested, washed, and resuspended in Tris-EDTA (TE) buffer with 100 mM LiAc. Fifty-microliter cell aliquots were incubated with heat-denatured salmon sperm DNA and 240 µl 50% polyethylene glycol (PEG) 3640, and then DNA was added. Samples were incubated at 30°C for 30 min. After addition of 45 µl DMSO, heat shock at 42°C for 15 min was performed. Centrifuged and resuspended cells were incubated for 2 h at 30°C and washed again before plating. Picked colonies were cultivated at 30°C overnight in 5% CO_2_ in minimal medium supplemented with histidine, methionine, and leucine. After inoculation of duplicates, one culture was transferred to air, while the second one was further incubated under 5% CO_2_. Fluorescence was detected with LSM 780 (Zeiss, Jena, Germany) at the time point of the switch from CO_2_ to air and after 60 and 120 min of incubation. GFP fluorescence of 50 cells per time point was measured using ZEN software (Zeiss), and mean fluorescence was calculated.

### Protein expression in *S. cerevisiae*.

Overexpression of Sc*CST6*-His and Sc*SCH9*-His in *S. cerevisiae* was achieved using the Sc*GAL1* promoter. Yeast cells were cultivated in minimal medium, supplemented with histidine, methionine, and leucine at 30°C overnight. Overexpression of Sc*CST6* and Sc*SCH9* was induced by a shift from minimal medium to YEP medium (1% yeast extract, 2% peptone) containing 2% raffinose and after overnight culture to YEP with 2% galactose for an additional 6 h. Cells were harvested, resuspended in yeast lysis buffer, and lysed with glass beads as described. Cell extracts were tested by Western blotting for overexpression.

### Immunoprecipitation.

Exponential *S. cerevisiae* WT or Sc*SCH9*-His-overexpressing cultures (50 ml) were harvested, resuspended in 500 µl immunoprecipitation (IP) buffer (20 mM sodium phosphate [pH 7], PhosStop, Complete protease inhibitor EDTA-free, PMSF), and lysed with glass beads as described. Extracts were incubated with either custom-made rabbit anti-Cst6, saturated with 5 µg 6×His tag peptide (Gentaur, Aachen, Germany), or anti-6×His antibody (GTX115045 [GeneTex, Hsinchu City, Taiwan]) overnight at 4°C. Fifty microliters of protein-G Sepharose beads (GE Healthcare) was washed with IP wash buffer (IP buffer plus 500 mM NaCl) and incubated with immunocomplexes for 1 h at 4°C. Beads were centrifuged at 2,500 rpm for 2 min, supernatant was taken off with a syringe and a 25-gauge needle, and beads were resuspended in wash buffer and incubated 1 min with shaking. This washing procedure was repeated four times. For coimmunoprecipitation (co-IP), beads were furthermore incubated with 20 µg recombinant ScSch9-His lysate for 3 h at 4°C. After extensive washing, beads were boiled in SDS sample buffer, subjected to 4 to 20% SDS-PAGE, and analyzed by Western blotting. Rabbit anti-ScCst6 (1:1,000) was used for detection of endogenous ScCst6 and fluorescein isothiocyanate (FITC)-labeled mouse anti-6×His (MA1-81891; 1:250 [Thermo Fisher Scientific]) for detection of 6×His-tagged ScSch9.

### Radioactive kinase assay.

ScSch9-His was immunoprecipitated from *S. cerevisiae* Sc*SCH9*-His-overexpressing culture. After washing of bead-bound immunocomplexes with IP wash buffer, an additional washing step with 1× kinase buffer (phosphate-buffered saline [PBS; pH 7.4], 4 mM MgCl, 10 mM DTT, 20% glycerol, 1 mM PMSF) was performed. One microgram of bacterially expressed and purified ScCst6-His was added to immunocomplexes in 50 µl kinase buffer. After addition of 100 µM ATP and 25 µCi [γ-^32^P]ATP (BLU002250UC; specific activity, 10 Ci/mmol [PerkinElmer, Waltham, MA]), reaction mixtures were incubated at 30°C for 30 min. The kinase reaction was stopped by heating at 95°C for 5 min in 4× SDS loading dye, and samples were subjected to 4 to 20% SDS-PAGE. Gels were dried, and radioactive incorporation was detected by autoradiography.

### LC-MS/MS analysis and protein database search.

To detect CO_2_-dependent phosphorylation in ScCst6, Sc*CST6-*overexpressing *S. cerevisiae* cultures were harvested, and ScCst6-His was purified using IMAC. For mass spectrometry analysis, in-gel digestion with trypsin-LysC, AspN, and GluC (all Promega, Madison, WI) or LysargiNase (50) and in-solution digestion with trypsin-LysC and GluC was performed. Peptides were extracted with trifluoroacetic acid and increasing concentrations of acetonitrile (50 to 90%) after in-gel digestion. Phosphopeptide enrichment was carried out using the TiO_2_ method (TiO_2_ Spin Tip sample prep kit, Protea Biosciences, Morgantown, WV), and the remaining peptides were purified with C_18_ minicolumns (C_18_ Spin Tip sample prep kit [Protea Biosciences, Morgantown, WV]). Dried peptides were solubilized in MS buffer (0.05% trifluoroacetic acid in 2% acetonitrile–98% H_2_O) and applied to LC-MS/MS analysis, carried out on an Ultimate 3000 nano-rapid-separation liquid chromatography (nano-RSLC) system coupled to a QExactive Plus mass spectrometer (both from Thermo Fisher Scientific). Thermo raw files were processed via the Proteome Discoverer (PD) v1.4.0.288. Tandem mass spectra were searched against the NCBI protein database of *S. cerevisiae* using the algorithms of Mascot v2.4.1 (Matrix Science, Inc., London, United Kingdom), Sequest HT, and MS Amanda. For further information, see Text S1 in the supplemental material.

10.1128/mBio.02211-16.7 TEXT S1  Supplemental methods. Download TEXT S1, PDF file, 0.1 MB. Copyright © 2017 Pohlers et al. 2017 Pohlers et al. This content is distributed under the terms of the Creative Commons Attribution 4.0 International license.

### Site-specific mutagenesis of Sc*CST6* and Sc*SCH9*.

Phosphoablative (S266A) and phosphomimetic (S266D) sequences of Sc*CST6* (GeneArt Gene Synthesis) were inserted in the pRS316 plasmid using PacI/AscI restriction sites. The Sc*CST6* promoter was cloned in pRS316 using SacI/PacI. After transformation of the *S. cerevisiae cst6*Δ mutant, Sc*NCE103* expression in 5% and 0.04% CO_2_ was measured as described and normalized to WT expression in 5% CO_2_. For construction of Sc*SCH9* with S/T-to-A mutation of all 6 TORC1 sites (*SCH9*^6A^), the sequence of *SCH9*^T570A, 6A^ was used as the template. A570 was remutated to T570 using QuikChange II site-directed mutagenesis kit (Agilent Technologies) with mutation-specific primers. The plasmid with the corrected sequence was checked via sequencing and used for transformation of the *sch9*Δ mutant.

### Construction of the *C. glabrata sch9*Δ mutant.

Deletion of Cg*SCH9* was achieved by replacement of the Cg*SCH9* open reading frame (ORF) with a *NAT1* cassette, which mediates resistance against nourseothricin. *NAT1* was amplified by PCR with EcoRI and SacI restriction sites and cloned into pBluescript. A homologous region upstream of the Cg*SCH9* ORF with a length of 1,000 bp was amplified by colony PCR and cloned into pBluescript using KpnI/XhoI. The same was done for a 1,000-bp fragment located downstream of Cg*SCH9* ORF using restriction sites SacII and SacI. The whole construct, the *NAT1* cassette flanked by homologous regions, was restricted with KpnI/SacI and purified in order to transform *C. glabrata*. AFG1, a strain lacking *LIG4*, was used for transformation because of its increased efficiency of homologous recombination (51). Transformation was performed using the lithium-acetate method, with cells plated on YPD with 100 µg/ml ClonNAT, and positive clones were selected. Integration of the *NAT1* cassette and deletion of Cg*SCH9* were checked by colony PCR with specific internal primers. Furthermore, deletion mutants were verified using external primers (see Fig. S3 in the supplemental material)[Supplementary-material textS2].

10.1128/mBio.02211-16.8 FIG S3  Verification of *C. glabrata sch9* deletion. (A) Insertion of *NAT1* and deletion of Cg*SCH9* were analyzed by colony PCR of the untransformed parental strain (AFG1) and *sch9* mutant. For amplification of *NAT1*, internal primers G1-CgSCH9 and X2-NAT1 were used for amplification of Cg*SCH9* G1-CgSCH9 and I2-CgSCH9. The strain AFG1 showed no signal for *NAT1* but did show signal for *SCH9*. In contrast, the *sch9* deletion mutant exhibited a PCR product for *NAT1* but not for *SCH9*. As a marker, the GeneRuler 1-kb DNA ladder (Thermo Fisher Scientific) was used in lane 1. (B) For verification of the correct position of *NAT1* cassette insertion, PCR with external primers G1-CgSCH9 and G4-CgSCH9 was used; these primers bind outside the inserted cassette. If the *SCH9* ORF is present, a PCR product of 4,385 bp occurs. If the *SCH9* ORF is replaced by *NAT1*, a shift to 3,463 bp is visible. As a marker, the GeneRuler 1-kb DNA ladder (Thermo Fisher Scientific) was used in lane 1. Download FIG S3, PDF file, 0.2 MB. Copyright © 2017 Pohlers et al. 2017 Pohlers et al. This content is distributed under the terms of the Creative Commons Attribution 4.0 International license.

10.1128/mBio.02211-16.9 TEXT S2  Supplemental references. Download TEXT S2, PDF file, 0.1 MB. Copyright © 2017 Pohlers et al. 2017 Pohlers et al. This content is distributed under the terms of the Creative Commons Attribution 4.0 International license.

### *NCE103* expression measurement of* C. glabrata* and *C. albicans.*

Measurement of Cg*NCE103* and Ca*NCE103* expression was performed according to the protocol for Sc*NCE103* expression described in the first section. As housekeeping genes, Cg*ACT1* for measurement of Cg*NCE103* and Ca*ACT1* for measurement of Ca*NCE103* were used.

### Statistical analysis.

Data are presented as arithmetic means ± standard deviation, and statistical significance (*P* < 0.05) was calculated using a two-sided *t* test for unpaired samples.
