# Supplementary figures and images for: Lipid Signaling via Pkh1/2 Regulates Fungal CO2 Sensing through the Kinase Sch9
Source: mBio. 2017 Jan 31;8(1):e02211-16. doi: 10.1128/mBio.02211-16 (PMC5263247; doi:10.1128/mBio.02211-16)

**A**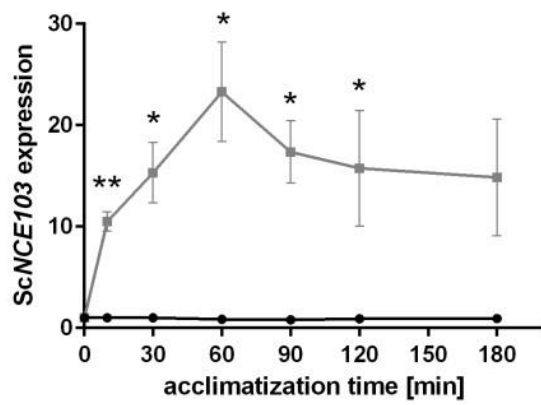

● 5% CO<sub>2</sub>    ■ air

**B**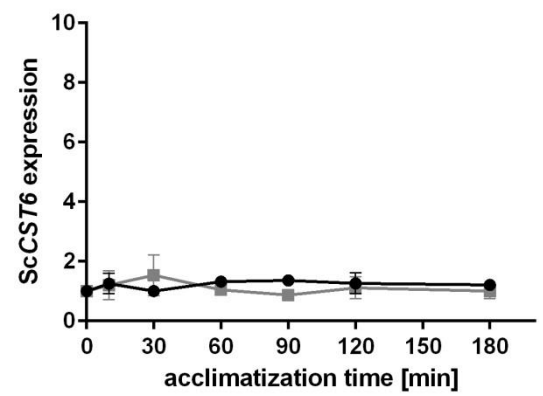

**Fig. S1 CO<sub>2</sub>-dependent time-course expression of *ScNCE103* and *ScCST6***

Supplement: FIG S1 [file mbo001173162sf1.pdf]

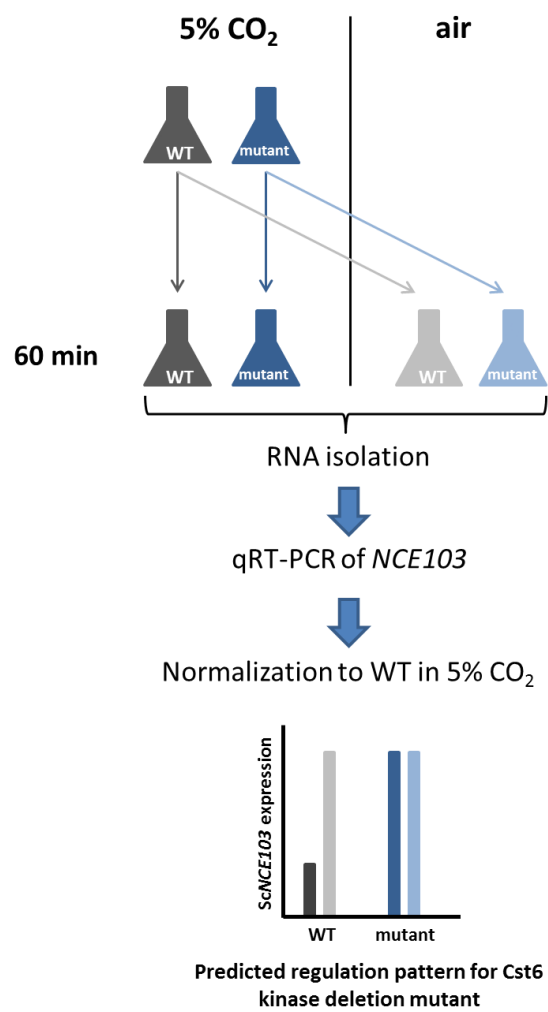

**Fig. S2 Workflow of mutant library screening for Cst6 kinase identification**

Supplement: FIG S2 [file mbo001173162sf2.pdf]

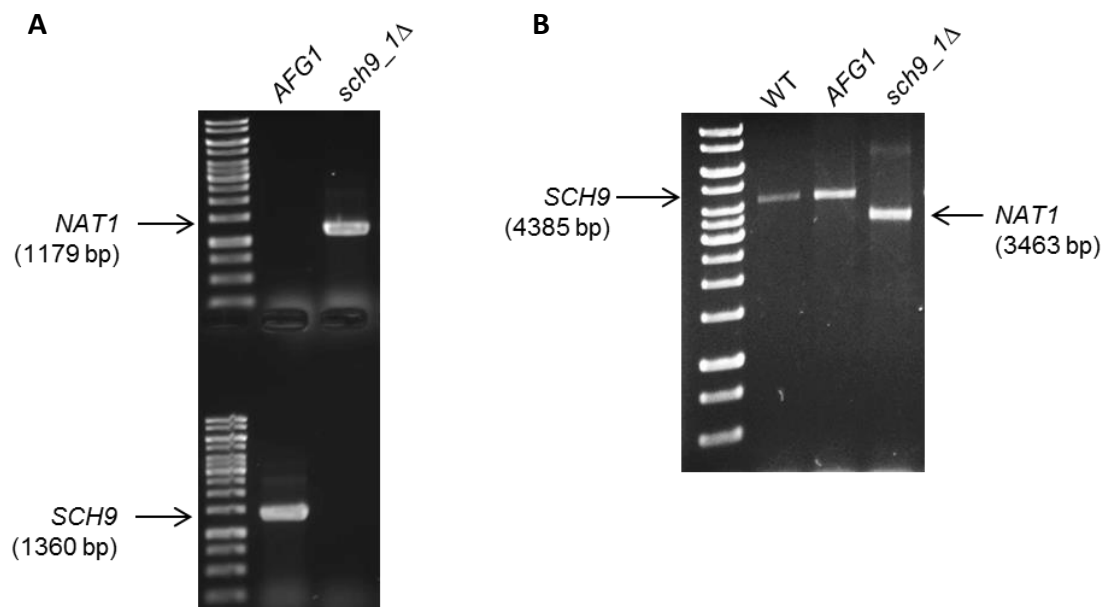

**Fig. S3 Verification of *C. glabrata* *sch9*Δ deletion**

Supplement: FIG S3 [file mbo001173162sf3.pdf]
