# Supplementary material for: Lipid Signaling via Pkh1/2 Regulates Fungal CO2 Sensing through the Kinase Sch9
Source: mBio. 2017 Jan 31;8(1):e02211-16. doi: 10.1128/mBio.02211-16 (PMC5263247; doi:10.1128/mBio.02211-16)
Supplement: TABLE S1 [file mbo001173162st1.pdf]

Table S1: Strains, plasmids and primers used in the study

S. cerevisiae mutant library			
**Name**	**Deletion**	**Genotype**	**Source/Reference**
wild type	-	BY4741, MAT a, his3Δ1; leu2Δ0,; met15Δ0; ura3Δ0	Euroscarf
akl1Δ	YBR059C	BY4741, MAT a, his3Δ1; leu2Δ0,; met15Δ0; ura3Δ0; YBR059c::kanMX4	Euroscarf
alk1Δ	YGL021W	BY4741, MAT a, his3Δ1; leu2Δ0,; met15Δ0; ura3Δ0; YGL021w::kanMX4	Euroscarf
alk2Δ	YBL009W	BY4741, MAT a, his3Δ1; leu2Δ0,; met15Δ0; ura3Δ0; YBL009w::kanMX4	Euroscarf
ark1Δ	YNL020C	BY4741, MAT a, his3Δ1; leu2Δ0,; met15Δ0; ura3Δ0; YNL020c::kanMX4	Euroscarf
atg1Δ	YGL180W	BY4741, MAT a, his3Δ1; leu2Δ0,; met15Δ0; ura3Δ0; YGL180w::kanMX4	Euroscarf
bck1Δ	YJL095W	BY4741, MAT a, his3Δ1; leu2Δ0,; met15Δ0; ura3Δ0; YJL095w::kanMX4	Euroscarf
bub1Δ	YGR188C	BY4741, MAT a, his3Δ1; leu2Δ0,; met15Δ0; ura3Δ0; YGR188c::kanMX4	Euroscarf
bud32Δ	YGR262C	BY4741, MAT a, his3Δ1; leu2Δ0,; met15Δ0; ura3Δ0; YGR262c::kanMX4	Euroscarf
cdc55Δ	YGL190C	BY4741, MAT a, his3Δ1; leu2Δ0,; met15Δ0; ura3Δ0; YGL190c::kanMX4	Euroscarf
chk1Δ	YBR274W	BY4741, MAT a, his3Δ1; leu2Δ0,; met15Δ0; ura3Δ0; YBR274w::kanMX4	Euroscarf
cka1Δ	YIL035C	BY4741, MAT a, his3Δ1; leu2Δ0,; met15Δ0; ura3Δ0; YIL035c::kanMX4	Euroscarf
cka2Δ	YOR061W	BY4741, MAT a, his3Δ1; leu2Δ0,; met15Δ0; ura3Δ0; YOR061w::kanMX4	Euroscarf
cla4Δ	YNL298W	BY4741, MAT a, his3Δ1; leu2Δ0,; met15Δ0; ura3Δ0; YNL298w::kanMX4	Euroscarf
cmk1Δ	YFR014C	BY4741, MAT a, his3Δ1; leu2Δ0,; met15Δ0; ura3Δ0; YFR014c::kanMX4	Euroscarf
cmk2Δ	YOL016C	BY4741, MAT a, his3Δ1; leu2Δ0,; met15Δ0; ura3Δ0; YOL016c::kanMX4	Euroscarf
cmp2Δ	YML057W	BY4741, MAT a, his3Δ1; leu2Δ0,; met15Δ0; ura3Δ0; YML057w::kanMX4	Euroscarf
cna1Δ	YLR433C	BY4741, MAT a, his3Δ1; leu2Δ0,; met15Δ0; ura3Δ0; YLR433c::kanMX4	Euroscarf
cnb1Δ	YKL190W	BY4741, MAT a, his3Δ1; leu2Δ0,; met15Δ0; ura3Δ0; YKL190w::kanMX4	Euroscarf
cst6Δ	YIL036W	BY4741, Mat a; his3D1; leu2D0; met15D0; ura3D0; YIL036w::kanMX4	Euroscarf
ctk1Δ	YKL139W	BY4741, MAT a, his3Δ1; leu2Δ0,; met15Δ0; ura3Δ0; YKL139w::kanMX4	Euroscarf
ctk2Δ	YJL006C	BY4741, MAT a, his3Δ1; leu2Δ0,; met15Δ0; ura3Δ0; YJL006c::kanMX4	Euroscarf
ctk3Δ	YML112W	BY4741, MAT a, his3Δ1; leu2Δ0,; met15Δ0; ura3Δ0; YML112w::kanMX4	Euroscarf
dbf2Δ	YGR092W	BY4741, MAT a, his3Δ1; leu2Δ0,; met15Δ0; ura3Δ0; YGR092w::kanMX4	Euroscarf
dbf20Δ	YPR111W	BY4742, MAT α, his3Δ1; leu2Δ0,; met15Δ0; ura3Δ0; YPR111w::kanMX4	Euroscarf
dcr2Δ	YLR361C	BY4741, MAT a, his3Δ1; leu2Δ0,; met15Δ0; ura3Δ0; YLR361c::kanMX4	Euroscarf

dun1Δ	YDL101C	BY4741, MAT a, his3Δ1; leu2Δ0,; met15Δ0; ura3Δ0; YDL101c::kanMX4	Euroscarf
elm1Δ	YKL048C	BY4741, MAT a, his3Δ1; leu2Δ0,; met15Δ0; ura3Δ0; YKL048c::kanMX4	Euroscarf
env7Δ	YPL236C	BY4741, MAT a, his3Δ1; leu2Δ0,; met15Δ0; ura3Δ0; YPL236c::kanMX4	Euroscarf
fpk1Δ	YNR047W	BY4741, MAT a, his3Δ1; leu2Δ0,; met15Δ0; ura3Δ0; YNR047w::kanMX4	Euroscarf
frk1Δ	YPL141C	BY4741, MAT a, his3Δ1; leu2Δ0,; met15Δ0; ura3Δ0; YPL141c::kanMX4	Euroscarf
fus3Δ	YBL016W	BY4741, MAT a, his3Δ1; leu2Δ0,; met15Δ0; ura3Δ0; YBL016w::kanMX4	Euroscarf
gal83Δ	YER027C	BY4741, MAT a, his3Δ1; leu2Δ0,; met15Δ0; ura3Δ0; YER027c::kanMX4	Euroscarf
gcn2Δ	YDR283C	BY4741, MAT a, his3Δ1; leu2Δ0,; met15Δ0; ura3Δ0; YDR283c::kanMX4	Euroscarf
gin4Δ	YDR507C	BY4741, MAT a, his3Δ1; leu2Δ0,; met15Δ0; ura3Δ0; YDR507c::kanMX4	Euroscarf
gpa2Δ	YER020W	BY4741, Mat a; his3D1; leu2D0; met15D0; ura3D0; YER020w::kanMX4	Euroscarf
hal5Δ	YJL165C	BY4741, MAT a, his3Δ1; leu2Δ0,; met15Δ0; ura3Δ0; YJL165c::kanMX4	Euroscarf
hog1Δ	YLR113W	BY4741, MAT a, his3Δ1; leu2Δ0,; met15Δ0; ura3Δ0; YLR113w::kanMX4	Euroscarf
hrk1Δ	YOR267C	BY4741, MAT a, his3Δ1; leu2Δ0,; met15Δ0; ura3Δ0; YOR267c::kanMX4	Euroscarf
hsl1Δ	YKL101W	BY4741, MAT a, his3Δ1; leu2Δ0,; met15Δ0; ura3Δ0; YKL101w::kanMX4	Euroscarf
ime2Δ	YJL106W	BY4741, MAT a, his3Δ1; leu2Δ0,; met15Δ0; ura3Δ0; YJL106w::kanMX4	Euroscarf
ire1Δ	YHR079C	BY4741, MAT a, his3Δ1; leu2Δ0,; met15Δ0; ura3Δ0; YHR079c::kanMX4	Euroscarf
isr1Δ	YPR106W	BY4741, MAT a, his3Δ1; leu2Δ0,; met15Δ0; ura3Δ0; YPR106w::kanMX4	Euroscarf
kcc4Δ	YCL024W	BY4741, MAT a, his3Δ1; leu2Δ0,; met15Δ0; ura3Δ0; YCL024w::kanMX4	Euroscarf
kdx1Δ	YKL161C	BY4741, MAT a, his3Δ1; leu2Δ0,; met15Δ0; ura3Δ0; YKL161c::kanMX4	Euroscarf
kin1Δ	YDR122W	BY4741, MAT a, his3Δ1; leu2Δ0,; met15Δ0; ura3Δ0; YDR122w::kanMX4	Euroscarf
kin2Δ	YLR096W	BY4741, MAT a, his3Δ1; leu2Δ0,; met15Δ0; ura3Δ0; YLR096w::kanMX4	Euroscarf
kin3Δ	YAR018C	BY4741, MAT a, his3Δ1; leu2Δ0,; met15Δ0; ura3Δ0; YAL018c::kanMX4	Euroscarf
kin4Δ	YOR233W	BY4741, MAT a, his3Δ1; leu2Δ0,; met15Δ0; ura3Δ0; YOR233w::kanMX4	Euroscarf
kin82Δ	YCR091W	BY4741, MAT a, his3Δ1; leu2Δ0,; met15Δ0; ura3Δ0; YCR091w::kanMX4	Euroscarf
kkq8Δ	YKL168C	BY4741, MAT a, his3Δ1; leu2Δ0,; met15Δ0; ura3Δ0; YKL168c::kanMX4	Euroscarf
kns1Δ	YLL019C	BY4741, MAT a, his3Δ1; leu2Δ0,; met15Δ0; ura3Δ0; YLL019c::kanMX4	Euroscarf
ksp1Δ	YHR082C	BY4741, MAT a, his3Δ1; leu2Δ0,; met15Δ0; ura3Δ0; YHR082c::kanMX4	Euroscarf
kss1Δ	YGR040W	BY4741, MAT a, his3Δ1; leu2Δ0,; met15Δ0; ura3Δ0; YGR040w::kanMX4	Euroscarf

lcb5Δ	YLR260W	BY4741, MAT a, his3Δ1; leu2Δ0,; met15Δ0; ura3Δ0; YLR260w::kanMX4	Euroscarf
ltp1Δ	YPR073C	BY4741, MAT a, his3Δ1; leu2Δ0,; met15Δ0; ura3Δ0; YPR073c::kanMX4	Euroscarf
mck1Δ	YNL307C	BY4741, MAT a, his3Δ1; leu2Δ0,; met15Δ0; ura3Δ0; YNL307c::kanMX4	Euroscarf
mek1Δ	YOR351C	BY4741, MAT a, his3Δ1; leu2Δ0,; met15Δ0; ura3Δ0; YOR351c::kanMX4	Euroscarf
mih1Δ	YMR036C	BY4741, MAT a, his3Δ1; leu2Δ0,; met15Δ0; ura3Δ0; YMR036c::kanMX4	Euroscarf
mkk1Δ	YOR231W	BY4741, MAT a, his3Δ1; leu2Δ0,; met15Δ0; ura3Δ0; YOR231w::kanMX4	Euroscarf
mkk2Δ	YPL140C	BY4741, MAT a, his3Δ1; leu2Δ0,; met15Δ0; ura3Δ0; YPL140c::kanMX4	Euroscarf
mrk1Δ	YDL079C	BY4741, MAT a, his3Δ1; leu2Δ0,; met15Δ0; ura3Δ0; YDL079c::kanMX4	Euroscarf
msg5Δ	YNL053W	BY4741, MAT a, his3Δ1; leu2Δ0,; met15Δ0; ura3Δ0; YNL053w::kanMX4	Euroscarf
nem1Δ	YHR004C	BY4741, MAT a, his3Δ1; leu2Δ0,; met15Δ0; ura3Δ0; YHR004c::kanMX4	Euroscarf
nnk1Δ	YKL171W	BY4741, MAT a, his3Δ1; leu2Δ0,; met15Δ0; ura3Δ0; YKL171w::kanMX4	Euroscarf
npr1Δ	YNL183C	BY4741, MAT a, his3Δ1; leu2Δ0,; met15Δ0; ura3Δ0; YNL183c::kanMX4	Euroscarf
oca1Δ	YNL099C	BY4741, MAT a, his3Δ1; leu2Δ0,; met15Δ0; ura3Δ0; YNL099c::kanMX4	Euroscarf
pbs2Δ	YJL128C	BY4741, MAT a, his3Δ1; leu2Δ0,; met15Δ0; ura3Δ0; YJL128c::kanMX4	Euroscarf
pho13Δ	YDL236W	BY4741, MAT a, his3Δ1; leu2Δ0,; met15Δ0; ura3Δ0; YDL236w::kanMX4	Euroscarf
pho85Δ	YPL031C	BY4741, MAT a, his3Δ1; leu2Δ0,; met15Δ0; ura3Δ0; YPL031c::kanMX4	Euroscarf
pkh1Δ	YDR490C	BY4741, MAT a, his3Δ1; leu2Δ0,; met15Δ0; ura3Δ0; YDR490c::kanMX4	Euroscarf
pkh2Δ	YOL100W	BY4741, MAT a, his3Δ1; leu2Δ0,; met15Δ0; ura3Δ0; YOL100w::kanMX4	Euroscarf
pkh3Δ	YDR466W	BY4741, MAT a, his3Δ1; leu2Δ0,; met15Δ0; ura3Δ0; YDR466w::kanMX4	Euroscarf
pkp1Δ	YIL042C	BY4741, MAT a, his3Δ1; leu2Δ0,; met15Δ0; ura3Δ0; YIL042c::kanMX4	Euroscarf
pkp2Δ	YGL059W	BY4741, MAT a, his3Δ1; leu2Δ0,; met15Δ0; ura3Δ0; YGL059w::kanMX4	Euroscarf
ppg1Δ	YNR032W	BY4741, MAT a, his3Δ1; leu2Δ0,; met15Δ0; ura3Δ0; YNR032w::kanMX4	Euroscarf
pph21Δ	YDL134C	BY4741, MAT a, his3Δ1; leu2Δ0,; met15Δ0; ura3Δ0; YDL134c::kanMX4	Euroscarf
pph22Δ	YDL188C	BY4741, MAT a, his3Δ1; leu2Δ0,; met15Δ0; ura3Δ0; YDL188c::kanMX4	Euroscarf
pph3Δ	YDR075W	BY4741, MAT a, his3Δ1; leu2Δ0,; met15Δ0; ura3Δ0; YDR075w::kanMX4	Euroscarf
ppq1Δ	YPL179W	BY4741, MAT a, his3Δ1; leu2Δ0,; met15Δ0; ura3Δ0; YPL179w::kanMX4	Euroscarf
pps1Δ	YBR276C	BY4741, MAT a, his3Δ1; leu2Δ0,; met15Δ0; ura3Δ0; YBR276c::kanMX4	Euroscarf
ppt1Δ	YGR123C	BY4741, MAT a, his3Δ1; leu2Δ0,; met15Δ0; ura3Δ0; YGR123c::kanMX4	Euroscarf

ppz1Δ	YML016C	BY4741, MAT a, his3Δ1; leu2Δ0,; met15Δ0; ura3Δ0; YML016c::kanMX4	Euroscarf
ppz2Δ	YDR436W	BY4741, MAT a, his3Δ1; leu2Δ0,; met15Δ0; ura3Δ0; YDR436w::kanMX4	Euroscarf
prk1Δ	YIL095W	BY4741, MAT a, his3Δ1; leu2Δ0,; met15Δ0; ura3Δ0; YIL095w::kanMX4	Euroscarf
prr1Δ	YKL116C	BY4741, MAT a, his3Δ1; leu2Δ0,; met15Δ0; ura3Δ0; YKL116c::kanMX4	Euroscarf
prr2Δ	YDL214C	BY4741, MAT a, his3Δ1; leu2Δ0,; met15Δ0; ura3Δ0; YDL214c::kanMX4	Euroscarf
psk1Δ	YAL017W	BY4741, MAT a, his3Δ1; leu2Δ0,; met15Δ0; ura3Δ0; YAL017w::kanMX4	Euroscarf
psk2Δ	YOL045W	BY4741, MAT a, his3Δ1; leu2Δ0,; met15Δ0; ura3Δ0; YOL045w::kanMX4	Euroscarf
psr1Δ	YLL010C	BY4741, MAT a, his3Δ1; leu2Δ0,; met15Δ0; ura3Δ0; YLL010c::kanMX4	Euroscarf
psr2Δ	YLR019W	BY4741, MAT a, his3Δ1; leu2Δ0,; met15Δ0; ura3Δ0; YLR019w::kanMX4	Euroscarf
ptc1Δ	YDL006W	BY4741, MAT a, his3Δ1; leu2Δ0,; met15Δ0; ura3Δ0; YDL006w::kanMX4	Euroscarf
ptc2Δ	YER089C	BY4741, MAT a, his3Δ1; leu2Δ0,; met15Δ0; ura3Δ0; YER089c::kanMX4	Euroscarf
ptc3Δ	YBL056W	BY4741, MAT a, his3Δ1; leu2Δ0,; met15Δ0; ura3Δ0; YBL056w::kanMX4	Euroscarf
ptc4Δ	YBR125C	BY4741, MAT a, his3Δ1; leu2Δ0,; met15Δ0; ura3Δ0; YBR125c::kanMX4	Euroscarf
ptc5Δ	YOR090C	BY4741, MAT a, his3Δ1; leu2Δ0,; met15Δ0; ura3Δ0; YOR090c::kanMX4	Euroscarf
ptc6Δ	YCR079W	BY4741, MAT a, his3Δ1; leu2Δ0,; met15Δ0; ura3Δ0; YCR079w::kanMX4	Euroscarf
ptc7Δ	YHR076W	BY4741, MAT a, his3Δ1; leu2Δ0,; met15Δ0; ura3Δ0; YHR076w::kanMX4	Euroscarf
ptk1Δ	YKL198C	BY4741, MAT a, his3Δ1; leu2Δ0,; met15Δ0; ura3Δ0; YKL198c::kanMX4	Euroscarf
ptk2Δ	YJR059W	BY4741, MAT a, his3Δ1; leu2Δ0,; met15Δ0; ura3Δ0; YJR059w::kanMX4	Euroscarf
ptp1Δ	YDL230W	BY4741, MAT a, his3Δ1; leu2Δ0,; met15Δ0; ura3Δ0; YDL230w::kanMX4	Euroscarf
ptp2Δ	YOR208W	BY4741, MAT a, his3Δ1; leu2Δ0,; met15Δ0; ura3Δ0; YOR208w::kanMX4	Euroscarf
ptp3Δ	YER075C	BY4741, MAT a, his3Δ1; leu2Δ0,; met15Δ0; ura3Δ0; YER075c::kanMX4	Euroscarf
rck1Δ	YGL158W	BY4741, MAT a, his3Δ1; leu2Δ0,; met15Δ0; ura3Δ0; YGL158w::kanMX4	Euroscarf
rck2Δ	YLR248W	BY4741, MAT a, his3Δ1; leu2Δ0,; met15Δ0; ura3Δ0; YLR248w::kanMX4	Euroscarf
rim11Δ	YMR139W	BY4741, MAT a, his3Δ1; leu2Δ0,; met15Δ0; ura3Δ0; YMR139w::kanMX4	Euroscarf
rim15Δ	YFL033C	BY4741, MAT a, his3Δ1; leu2Δ0,; met15Δ0; ura3Δ0; YFL033c::kanMX4	Euroscarf
rtk1Δ	YDL025C	BY4741, MAT a, his3Δ1; leu2Δ0,; met15Δ0; ura3Δ0; YDL025c::kanMX4	Euroscarf
rtr1Δ	YER139C	BY4741, MAT a, his3Δ1; leu2Δ0,; met15Δ0; ura3Δ0; YER139c::kanMX4	Euroscarf
rts1Δ	YOR014W	BY4741, MAT a, his3Δ1; leu2Δ0,; met15Δ0; ura3Δ0; YOR014w::kanMX4	Euroscarf

sak1Δ	YER129W	BY4741, MAT a, his3Δ1; leu2Δ0,; met15Δ0; ura3Δ0; YER129w::kanMX4	Euroscarf
sap155Δ	YFR040W	BY4741, MAT a, his3Δ1; leu2Δ0,; met15Δ0; ura3Δ0; YFR040w::kanMX4	Euroscarf
sap185Δ	YJL098W	BY4741, MAT a, his3Δ1; leu2Δ0,; met15Δ0; ura3Δ0; YJL098w::kanMX4	Euroscarf
sap4Δ	YGL229C	BY4741, MAT a, his3Δ1; leu2Δ0,; met15Δ0; ura3Δ0; YGL229c::kanMX4	Euroscarf
sat4Δ	YCR008W	BY4741, MAT a, his3Δ1; leu2Δ0,; met15Δ0; ura3Δ0; YCR008w::kanMX4	Euroscarf
sch9Δ	YHR205W	BY4741, MAT a, his3Δ1; leu2Δ0,; met15Δ0; ura3Δ0; YHR205w::kanMX4	Euroscarf
sdp1Δ	YIL113W	BY4741, MAT a, his3Δ1; leu2Δ0,; met15Δ0; ura3Δ0; YIL113w::kanMX4	Euroscarf
sip1Δ	YDR422C	BY4741, MAT a, his3Δ1; leu2Δ0,; met15Δ0; ura3Δ0; YDR422c::kanMX4	Euroscarf
sip2Δ	YGL208W	BY4741, MAT a, his3Δ1; leu2Δ0,; met15Δ0; ura3Δ0; YGL208w::kanMX4	Euroscarf
siw14Δ	YNL032W	BY4741, MAT a, his3Δ1; leu2Δ0,; met15Δ0; ura3Δ0; YNL032w::kanMX4	Euroscarf
skm1Δ	YOL113W	BY4741, MAT a, his3Δ1; leu2Δ0,; met15Δ0; ura3Δ0; YOL113w::kanMX4	Euroscarf
sks1Δ	YPL026C	BY4741, MAT a, his3Δ1; leu2Δ0,; met15Δ0; ura3Δ0; YPL026c::kanMX4	Euroscarf
sky1Δ	YMR216C	BY4741, MAT a, his3Δ1; leu2Δ0,; met15Δ0; ura3Δ0; YMR216c::kanMX4	Euroscarf
slt2Δ	YHR030C	BY4741, MAT a, his3Δ1; leu2Δ0,; met15Δ0; ura3Δ0; YHR030c::kanMX4	Euroscarf
smk1Δ	YPR054W	BY4741, MAT a, his3Δ1; leu2Δ0,; met15Δ0; ura3Δ0; YPR054w::kanMX4	Euroscarf
snf1Δ	YDR477W	BY4742, MAT α, his3Δ1; leu2Δ0,; met15Δ0; ura3Δ0; YDR477w::kanMX4	Euroscarf
snf4Δ	YGL115W	BY4741, MAT a, his3Δ1; leu2Δ0,; met15Δ0; ura3Δ0; YGL115w::kanMX4	Euroscarf
spo7Δ	YAL009W	BY4741, MAT a, his3Δ1; leu2Δ0,; met15Δ0; ura3Δ0; YAL009w::kanMX4	Euroscarf
ssk2Δ	YNR031C	BY4741, MAT a, his3Δ1; leu2Δ0,; met15Δ0; ura3Δ0; YNR031c::kanMX4	Euroscarf
ssk22Δ	YCR073C	BY4741, MAT a, his3Δ1; leu2Δ0,; met15Δ0; ura3Δ0; YCR073c::kanMX4	Euroscarf
ssn3Δ	YPL042C	BY4741, MAT a, his3Δ1; leu2Δ0,; met15Δ0; ura3Δ0; YPL042c::kanMX4	Euroscarf
ste11Δ	YLR362W	BY4741, MAT a, his3Δ1; leu2Δ0,; met15Δ0; ura3Δ0; YLR362w::kanMX4	Euroscarf
ste20Δ	YHL007C	BY4741, MAT a, his3Δ1; leu2Δ0,; met15Δ0; ura3Δ0; YHL007c::kanMX4	Euroscarf
ste7Δ	YDL159W	BY4741, MAT a, his3Δ1; leu2Δ0,; met15Δ0; ura3Δ0; YDL159w::kanMX4	Euroscarf
swe1Δ	YJL187C	BY4741, MAT a, his3Δ1; leu2Δ0,; met15Δ0; ura3Δ0; YJL187c::kanMX4	Euroscarf
tda1Δ	YMR291W	BY4741, MAT a, his3Δ1; leu2Δ0,; met15Δ0; ura3Δ0; YMR291w::kanMX4	Euroscarf
tel1Δ	YBL088C	BY4741, MAT a, his3Δ1; leu2Δ0,; met15Δ0; ura3Δ0; YBL088c::kanMX4	Euroscarf
tor1Δ	YJR066W	BY4741, MAT a, his3Δ1; leu2Δ0,; met15Δ0; ura3Δ0; YJR066w::kanMX4	Euroscarf

tos3Δ	YGL179C	BY4741, MAT a, his3Δ1; leu2Δ0,; met15Δ0; ura3Δ0; YGL179c::kanMX4	Euroscarf
tpd3Δ	YAL016W	BY4741, MAT a, his3Δ1; leu2Δ0,; met15Δ0; ura3Δ0; YAL016w::kanMX4	Euroscarf
tpk1Δ	YJL164C	BY4741, MAT a, his3Δ1; leu2Δ0,; met15Δ0; ura3Δ0; YJL164c::kanMX4	Euroscarf
tpk2Δ	YPL203W	BY4741, MAT a, his3Δ1; leu2Δ0,; met15Δ0; ura3Δ0; YPL203w::kanMX4	Euroscarf
tpk3Δ	YKL166C	BY4741, MAT a, his3Δ1; leu2Δ0,; met15Δ0; ura3Δ0; YKL166c::kanMX4	Euroscarf
vhs1Δ	YDR247W	BY4741, MAT a, his3Δ1; leu2Δ0,; met15Δ0; ura3Δ0; YDR247w::kanMX4	Euroscarf
yak1Δ	YJL141C	BY4741, MAT a, his3Δ1; leu2Δ0,; met15Δ0; ura3Δ0; YJL141c::kanMX4	Euroscarf
ych1Δ	YGR203W	BY4741, MAT a, his3Δ1; leu2Δ0,; met15Δ0; ura3Δ0; YGR203w::kanMX4	Euroscarf
yck1Δ	YHR135C	BY4741, MAT a, his3Δ1; leu2Δ0,; met15Δ0; ura3Δ0; YHR135c::kanMX4	Euroscarf
yck2Δ	YNL154C	BY4741, MAT a, his3Δ1; leu2Δ0,; met15Δ0; ura3Δ0; YNL154c::kanMX4	Euroscarf
yck3Δ	YER123W	BY4741, MAT a, his3Δ1; leu2Δ0,; met15Δ0; ura3Δ0; YER123w::kanMX4	Euroscarf
ygk3Δ	YOL128C	BY4741, MAT a, his3Δ1; leu2Δ0,; met15Δ0; ura3Δ0; YOL128c::kanMX4	Euroscarf
ypk1Δ	YKL126W	BY4741, MAT a, his3Δ1; leu2Δ0,; met15Δ0; ura3Δ0; YKL126w::kanMX4	Euroscarf
ypk2Δ	YMR104C	BY4741, MAT a, his3Δ1; leu2Δ0,; met15Δ0; ura3Δ0; YMR104c::kanMX4	Euroscarf
ypk3Δ	YBR028C	BY4741, MAT a, his3Δ1; leu2Δ0,; met15Δ0; ura3Δ0; YBR028c::kanMX4	Euroscarf
YPL150WΔ	YPL150W	BY4741, MAT a, his3Δ1; leu2Δ0,; met15Δ0; ura3Δ0; YPL150w::kanMX4	Euroscarf
yvh1Δ	YIR026C	BY4741, MAT a, his3Δ1; leu2Δ0,; met15Δ0; ura3Δ0; YIR026c::kanMX4	Euroscarf

other *S. cerevisiae* strains

Name	Deletion	Genotype	Source/Reference
BY4741 + SCH9-His	-	BY4741 + pRS316-promScGAL1-ScSch9-His (URA3 positive)	this study
BY4741 + promNCE103-GFP	-	BY4741 + pRS316-pScNCE103GFP (URA3 positive)	this study
sch9Δ + promNCE103-GFP	YHR205W	YHR205WΔ + pRS316-pScNCE103GFP (URA3 positive)	this study
cst6Δ + promNCE103-GFP	YIL036W	YIL036WΔ + pRS316-pScNCE103GFP (URA3 positive)	this study
cst6Δ + CST6	YIL036W	YIL036WΔ + pRS316-promScCST6-ScCST6-His (URA3 positive)	this study
cst6Δ + CST6 (S266A)	YIL036W	YIL036WΔ + pRS316-promScCST6-ScCST6 (S266A) (URA3 positive)	this study
cst6Δ + CST6 (S266D)	YIL036W	YIL036WΔ + pRS316-promScCST6-ScCST6 (S266D) (URA3 positive)	this study
sch9Δ + SCH9	YHR205W	YHR205WΔ + pRS416-promScSCH9-ScSCH9-3HA (URA3 positive)	this study

sch9Δ + SCH9 (T570A)	YHR205W	YHR205WΔ + pRS416-promScSCH9-ScSCH9-3HA (T570A) (URA3 positive)	this study
sch9Δ + SCH9 (6A)	YHR205W	YHR205WΔ + pRS416-promScSCH9-ScSCH9-3HA (S711A, T723A, S726A, T737A, S758A, S765A) (URA3 positive)	this study
sch9Δ + SCH9 (T570A, 6A)	YHR205W	YHR205WΔ + pRS416-promScSCH9-ScSCH9-3HA (T570A, S711A, T723A, S726A, T737A, S758A, S765A) (URA3 positive)	this study
15 Dau	-	*MATa leu2 ura3 trp1 his2 ade1*	P. Van Dijck / Inagaki et al., 1999 (1)
pkh1^{ts} pkh2Δ	YOL100W	15 Dau *pkh1::PKH1D398G pkh2::LEU2*	P. Van Dijck / Inagaki et al., 1999 (1)

C. glabrata strains

**Name**	**Deletion**	**Genotype**	**Source/Reference**
ATCC 2001	-	wild type	F. Mühlschlegel / Kitada et al., 1995 (2)
rca1Δ	RCA1	ATCC 2001, rca1Δ::TRP1, trp1, Δ ura3	F. Mühlschlegel / Cottier et al., 2013
AFG1	LIG4	lig4::HIS1, trp1Δ isolate A	P. Van Dijck / Cen et al., 2015
sch9Δ	LIG4, SCH9	lig4::HIS1, trp1Δ, sch9::NAT1	this study

C. albicans strains

**Name**	**Deletion**	**Genotype**	**Source/Reference**
SC5314	-	wild type	B. Hube
rca1ΔY	RCA1	leu2Δ/leu2Δ his1Δ/his1Δ arg4Δ/arg4Δ URA3/ura3Δ::imm434 IRO1/iro1Δ::imm434 rca1::LEU2/rca1::HIS1 clone Y	B. Hube
sch9Δ	SCH9	CAI4, sch9Δ	J. Ernst / Stichternoth et al., 2011

E. coli strains

**Name**	**Genotype**	**Source/Reference**
BL21DE3	*fhuA2 [lon] ompT gal (λ DE3) [dcm] ΔhsdS, λ DE3 = λ sBamHIo ΔEcoRI-B int::(lacI::PlacUV5::T7 gene1) i21 Δnin5*	New England Biolabs
C2523H	*fhuA2 [lon] ompT gal sulA11 R(mcr-73::miniTn10*--Tet^S*)2 [dcm] R(zgb-210::Tn10*--Tet^S*) endA1 Δ(mcrC-mrr)114::IS10*	New England Biolabs
Rosetta	F$^-$ *ompT hsdS*$_B$(r$_B^-$ m$_B^-$) *gal dcm* (DE3) pRARE (Cam^R)	Novagen, Merck Millipore

Plasmids

**Name**	**Source/Reference**
p41-ScNce103-His	this study
p41-ScCst6-His	this study
p41-ScSch9-His	this study
pRS316-promScGAL1-ScCST6-His	this study
pRS316-promScGAL1-ScSch9-His	this study
pRS316-pScNCE103+GFP	this study
pRS316-promScCST6-ScCST6-His	this study

pRS316-promScCST6-ScCST6 (S266A)	this study
pRS316-promScCST6-ScCST6 (S266D)	this study
pRS416-promScSCH9-ScSCH9-3HA	R. Loewith / Urban et al., 2007
pRS416-promScSCH9-ScSCH9-3HA (T570A)	R. Loewith / Urban et al., 2007
pGEX-6P-1 ScSCH9-3HA (S711A, T723A, S726A, T737A, S758A, S765A)	R. Loewith / Urban et al., 2007
pRS416-promScSCH9-ScSCH9-3HA (S711A, T723A, S726A, T737A, S758A, S765A)	this study
pRS416-promScSCH9-ScSCH9-3HA (T570A, S711A, T723A, S726A, T737A, S758A, S765A)	R. Loewith / Urban et al., 2007
pBluescript	Agilent Technologies
pMA-T-ScNce103-His	GeneArt Gene Synthesis, Thermo Fisher Scientific
pMK-RQ-ScCst6-His	GeneArt Gene Synthesis, Thermo Fisher Scientific
pMK-RQ-ScSch9-His	GeneArt Gene Synthesis, Thermo Fisher Scientific

Primer for qRT-PCR

Name	Sequence
ScNCE103 fwd	GATATTGACACCATGTACCATGA
ScNCE103 rev	TGTAAGTGCTAACTGTTTGCA
ScACT1 fwd	TTCCCAGGTATTGCCGA
ScACT1 rev	TGTGGTGAACGATAGATGGA
CgNCE103 fwd	CATGAATCTGAGCTGAACCA
CgNCE103 rev	TGATTGCCATAGACCTCTCTTA
CgACT1 fwd	ATCATTGCTCCACCAGAGA
CgACT1 rev	TGTGGTGAACAATGGATGG
CaNCE103 fwd	TCCAGTTAGACATATTCGTGCTG
CaNCE103 rev	TGAGGGTTATATTCTTCTTCATCATG
CaACT1 fwd	TCAGACCAGCTGATTTAGGTTTG
CaACT1 rev	GTGAACAATGGATGGACCAG

Primer for protein expression (*E. coli*)

Name	Sequence
fw ScNce103 Exp p41 VspI	GCGCAAATTAATATGAGCGCTACCGAATC
rv ScNce103 Exp p41	GGTCAAGCTTATCAATGATGGTGATGATGGTGTTTTGGGGTAACTTTTGTGTAAG
rv ScCst6 Exp p41	GGTCAAGCTTATCAATGATGGTGATGATGGTGTTTTATCTTTTCAGAATTGGGTAATG
fw ScCst6 Exp p41	GCGCAACATATGTTTACTGGTCAGGAGTATCATTC
p41-SCH9-fwd	AAGACAGAGCGAAATTCTATATCG
p41-SCH9-rev	AGTTTGTTCGGCCGGATCCGT

Primer for protein expression (*S. cerevisiae*)

Name	Sequence
5´-ScGAL1prom-Sac1	ATGCGAGCTCTTATATTGAATTTTCAAAAATTCTTACTTTTTTTTTGGAT
3´-ScGAL1prom-Pac1	ACATTTAATTAATATAGTTTTTTCTCCTTGACGTTAAAGTATAG
fw ScCst6 in pRS316	TAACATTAATTAAATGTTTACTGGTCAGGAGTATC
rv ScCst6 in pRS316	GAAGTGGCGCGCCTTATTTTATCTTTTCAGAATTGGGTAATG
pRS316 SCH9-Pac1 fwd	TATATTAATTAACATATGATGAATTTTTTTACATCAAAATCGTC
pRS316 SCH9-Asc1 rev	AAGTGGCGCGCCAAGCTTATTATCAATG
5’pRS316-veri	CATTCGCCATTCAGGCTGCG
3’pRS316-veri	TCGAGGTCGACGGTATCGAT
5’SCH9 T570 sequ	ACATTCTACTCGATGCC
3’SCH9 6A sequ	ATCTGCGAATGGCTGTTG
**Primer for site-specific mutagenesis (*S. cerevisiae*)**	
**Name**	**Sequence**
5’SCH9 A570T	CTTGAAGGATAGAACAAACACATTTTGCGGCACCACGG
3’SCH9 A570T	CCGTGGTGCCGCAAAATGTGTTTGTTCTATCCTTCAAG
**Primer for *C. glabrata SCH9* deletion**	
**Name**	**Sequence**
5´NAT1-EcoR1	TAT CGA ATT CCA TAG CTT CAA AAT GTT TCT
3´NAT1-Pst1	CGGGCTGCAGGCAAATTAAAGCC
5´CgSCH9prom-Kpn1	TAAG GGTACC AAAAGTTGGAACCTGTAG
3´CgSCH9prom-Xho1	TATA CTCGAG ATAAATGTTTATTGAAGTGTGAAC
5´CgSCH9term-Sac2	TGAT CCGCGG ATTTGTCTTTCATTTTGGTT
3´CgSCH9term-Sac1	AGCA GAGCTCAAGGTGTTACTAATTCCATATTT
G1-CgSCH9	AAGAAACTGTTATTGTATATGTTGTCCCTT
G4-CgSCH9	CTGCATCTAGACGGAGTTTGACTCTT
I2-CgSCH9	TATAGCGGGCTTATCATTAACACCAATA
X2-NAT1	CTGTGCTTGGGTGTTTTGAAGTGGTAC
