# Supplementary material for: Lipid Signaling via Pkh1/2 Regulates Fungal CO2 Sensing through the Kinase Sch9
Source: mBio. 2017 Jan 31;8(1):e02211-16. doi: 10.1128/mBio.02211-16 (PMC5263247; doi:10.1128/mBio.02211-16)
Supplement: TABLE S2 [file mbo001173162st2.pdf]

Table S2: Mean Sc*NCE103* expression and standard deviation (SD) of *S. cerevisiae* kinase/phosphatase mutants in changing CO_2 environment

	NCE103 ^{CO2}	SD ^{CO2}	*NCE103* ^{air}	SD ^{air}	fold change air / CO_2	SD fold change
**wild type**	1,00		5,64	2,63	5,64	2,63
***akl1*Δ**	1,54	0,90	5,81	2,48	5,13	4,62
***alk1*Δ**	0,67	0,35	1,89	0,16	3,22	1,28
***alk2*Δ**	1,16	0,11	5,22	4,17	4,42	3,28
***ark1*Δ**	1,66	0,92	3,31	2,67	2,87	3,19
***atg1*Δ**	0,77	0,14	2,84	1,93	3,51	1,85
***bck1*Δ**	1,42	0,78	5,36	1,30	4,16	1,38
***bub1*Δ**	1,52	0,64	13,11	3,99	9,14	2,78
***bud32*Δ**	3,38	0,79	36,54	8,97	10,85	1,55
***cdc55*Δ**	1,31	0,34	12,32	2,36	9,86	3,20
***chk1*Δ**	1,12	0,03	3,36	1,79	2,99	1,51
***cka1*Δ**	0,95	0,31	2,86	1,44	3,44	2,57
***cka2*Δ**	0,86	0,03	1,96	0,44	2,27	0,42
***cla4*Δ**	0,76	0,59	5,52	0,14	10,21	7,72
***cmk1*Δ**	0,75	0,21	2,64	1,09	4,05	2,96
***cmk2*Δ**	1,06	0,13	5,74	2,01	5,55	2,57
***cmp2*Δ**	1,49	0,45	3,33	1,61	2,16	0,42
***cna1*Δ**	1,35	0,68	3,94	3,07	2,68	0,93
***cnb1*Δ**	1,61	0,09	6,36	1,48	3,92	0,69
***cst6*Δ**	0,97	0,20	2,70	1,65	2,75	1,45
***ctk1*Δ**	1,44	0,74	7,65	4,14	5,35	2,57
***ctk2*Δ**	1,71	0,48	7,30	2,78	4,35	1,47
***ctk3*Δ**	1,57	0,38	5,94	2,15	3,80	1,30
***dbf2*Δ**	1,34	0,32	5,88	1,81	4,34	0,32
***dbf20*Δ**	1,08	0,07	4,50	1,31	4,15	0,95
***dcr2*Δ**	0,88	0,25	3,01	0,70	3,46	0,20
***dun1*Δ**	0,79	0,27	3,01	1,40	3,76	1,35
***elm1*Δ**	1,24	0,08	3,76	0,50	3,06	0,60
***env7*Δ**	1,10	0,09	5,79	2,29	5,37	2,53
***fpk1*Δ**	1,07	0,57	2,64	0,79	2,66	0,68
***frk1*Δ**	1,01	0,10	4,63	0,61	4,58	0,16
***fus3*Δ**	1,34	0,32	4,35	0,08	3,34	0,75
***gal83*Δ**	0,81	0,37	2,51	0,34	3,37	1,13
***gcn2*Δ**	1,29	0,17	3,94	1,01	3,02	0,38
***gin4*Δ**	0,96	0,38	3,45	1,66	3,53	0,33
***gpa2*Δ**	0,84	0,01	5,44	5,04	6,53	6,12
***hal5*Δ**	1,21	0,37	2,27	1,13	1,97	1,08
***hog1*Δ**	1,07	0,34	4,18	1,07	3,91	1,41
***hrk1*Δ**	0,65	0,13	2,72	0,88	4,14	0,55
***hsl1*Δ**	1,65	0,19	4,02	2,59	2,54	1,87
***ime2*Δ**	1,00	0,45	2,97	0,38	3,80	2,79
***ire1*Δ**	1,02	0,58	2,59	0,13	3,07	1,88

isr1Δ	0,79	0,26	4,16	2,01	6,03	4,56
kcc4Δ	0,92	0,18	3,60	0,41	3,94	0,33
kdx1Δ	1,62	0,06	4,20	2,51	2,57	1,46
kin1Δ	0,82	0,31	3,16	2,01	3,99	2,23
kin2Δ	1,17	0,00	3,34	1,73	2,85	1,48
kin3Δ	1,53	0,42	4,12	0,24	2,82	0,94
kin4Δ	1,28	0,04	5,28	2,04	4,09	1,47
kin82Δ	0,93	0,14	3,21	0,59	3,45	0,12
kkq8Δ	0,84	0,43	4,95	2,59	5,84	0,09
kns1Δ	1,03	0,39	3,88	0,21	4,03	1,32
ksp1Δ	1,18	0,71	3,08	0,41	3,05	1,49
kss1Δ	0,88	0,50	1,71	0,41	2,15	0,74
lcb5Δ	0,78	0,31	2,86	1,38	3,60	0,33
ltp1Δ	1,09	0,05	3,68	0,54	3,35	0,33
mck1Δ	1,73	0,86	6,47	4,03	4,24	3,16
mek1Δ	1,21	0,01	3,92	0,82	3,23	0,64
mih1Δ	1,45	0,57	3,76	0,51	2,98	1,45
mkk1Δ	1,10	0,86	4,56	3,60	4,14	0,04
mkk2Δ	0,94	0,23	4,50	3,43	5,37	4,95
mrk1Δ	0,58	0,19	2,81	1,98	4,70	2,58
msg5Δ	1,11	0,30	3,60	0,37	3,31	0,56
nem1Δ	0,97	0,39	3,79	0,37	4,32	2,11
nnk1Δ	0,83	0,20	2,32	0,43	2,82	0,15
npr1Δ	0,89	0,50	3,81	0,22	5,01	2,56
oca1Δ	1,57	0,47	5,72	2,54	3,55	0,55
pbs2Δ	1,23	0,65	2,66	2,36	2,30	0,60
pho13Δ	1,34	0,60	5,06	0,99	4,67	3,24
pho85Δ	1,21	0,08	5,77	2,47	4,70	1,72
pkh1Δ	0,85	0,39	2,09	0,83	2,49	0,18
pkh2Δ	0,85	0,02	2,71	0,08	3,20	0,00
pkh3Δ	0,69	0,48	1,84	0,47	3,21	1,57
pkp1Δ	1,17	0,26	4,33	3,49	3,46	2,10
pkp2Δ	1,54	1,29	2,96	1,20	2,74	1,81
ppg1Δ	0,96	0,20	3,48	0,14	3,71	0,65
pph21Δ	1,14	0,68	5,64	4,49	4,57	1,22
pph22Δ	1,69	0,75	6,10	1,85	4,28	3,01
pph3Δ	0,89	0,16	3,06	1,91	3,72	2,84
ppq1Δ	1,01	0,05	2,88	0,18	2,86	0,34
pps1Δ	0,73	0,18	4,58	0,58	6,41	0,78
ppt1Δ	1,05	0,03	3,81	0,59	3,62	0,65
ppz1Δ	1,53	0,73	4,20	1,22	2,89	0,59
ppz2Δ	1,13	0,18	5,11	3,86	4,31	2,73
prk1Δ	0,93	0,54	2,70	0,02	3,47	2,00
prr1Δ	1,46	0,58	4,05	2,83	2,71	1,19
prr2Δ	1,03	0,21	3,24	1,31	3,07	0,66
psk1Δ	0,90	0,26	3,91	0,29	4,49	0,96
psk2Δ	0,80	0,02	3,41	1,77	4,30	2,32

psr1Δ	0,98	0,60	4,31	1,07	5,01	1,98
psr2Δ	1,35	0,44	3,54	1,54	2,58	0,30
ptc1Δ	1,31	0,74	7,53	3,47	7,57	6,60
ptc2Δ	0,96	0,25	1,74	0,88	1,80	0,78
ptc3Δ	1,58	0,42	4,52	0,53	2,91	0,44
ptc4Δ	1,12	0,06	3,88	0,49	3,47	0,25
ptc5Δ	0,84	0,03	3,30	0,88	3,94	1,20
ptc6Δ	1,15	0,27	5,79	3,76	4,74	1,97
ptc7Δ	1,30	0,62	2,77	0,72	2,33	0,92
ptk1Δ	0,94	0,23	2,41	1,49	2,43	0,97
ptk2Δ	2,44	2,09	8,65	9,08	2,98	0,97
ptp1Δ	2,07	0,72	3,99	2,64	2,13	1,51
ptp2Δ	0,72	0,25	3,45	0,77	5,36	2,98
ptp3Δ	1,05	0,25	3,91	0,74	3,92	1,66
rck1Δ	1,04	0,26	2,66	0,16	2,65	0,82
rck2Δ	0,96	0,28	3,24	0,30	3,47	0,71
rim11Δ	1,78	0,51	3,24	0,24	1,91	0,68
rim15Δ	1,03	0,34	4,30	2,72	3,96	1,34
rtk1Δ	0,82	0,33	3,92	1,23	5,50	3,69
rtr1Δ	0,90	0,44	9,62	5,15	10,57	0,61
rts1Δ	1,55	0,79	3,56	0,90	2,47	0,68
sak1Δ	0,56	0,04	3,13	1,27	5,50	1,88
sap155Δ	1,17	0,08	7,61	0,78	6,52	0,22
sap185Δ	1,30	0,14	4,06	0,58	3,13	0,11
sap4Δ	1,71	0,46	6,54	6,03	3,39	2,45
sat4Δ	1,01	0,20	2,08	0,98	2,22	1,27
sch9Δ	3,55	1,55	6,12	2,98	1,91	0,70
sdp1Δ	1,26	0,25	2,45	0,30	1,95	0,14
sip1Δ	1,06	0,27	3,64	3,08	3,15	2,08
sip2Δ	0,91	0,33	2,41	0,48	2,93	1,58
siw14Δ	1,25	0,14	4,85	5,79	3,64	4,23
skm1Δ	1,10	0,08	3,08	0,67	2,80	0,42
sks1Δ	1,02	0,01	3,41	0,32	3,32	0,29
sky1Δ	1,08	0,80	2,07	2,21	1,76	0,78
slt2Δ	0,93	0,05	4,92	1,64	5,33	1,95
smk1Δ	1,33	0,23	4,58	2,04	3,63	2,17
snf1Δ	1,26	0,49	4,62	1,54	5,31	2,82
snf4Δ	0,98	0,37	2,99	1,42	3,57	2,80
spo7Δ	0,94	0,20	7,33	1,39	7,87	0,44
ssk2Δ	1,73	1,33	5,17	2,16	3,52	1,06
ssk22Δ	0,55	0,20	2,04	0,41	3,82	0,62
ssn3Δ	1,35	0,11	4,03	0,49	2,97	0,12
ste11Δ	1,20	0,84	4,51	3,27	4,01	1,58
ste20Δ	1,98	1,50	3,03	2,43	1,56	0,65
ste7Δ	1,79	0,74	5,21	4,14	2,93	1,69
swe1Δ	1,18	0,20	5,90	5,11	4,70	3,55
tda1Δ	1,73	0,19	4,17	1,67	2,47	1,23

***tel1*Δ**	1,35	0,78	2,68	0,13	2,35	1,27
***tor1*Δ**	1,51	0,37	10,48	4,96	7,78	5,27
***tos3*Δ**	0,91	0,72	2,04	0,17	3,16	2,31
***tpd3*Δ**	2,16	0,65	9,60	2,58	4,80	1,85
***tpk1*Δ**	1,30	0,12	2,65	0,76	2,07	0,77
***tpk2*Δ**	0,86	0,39	2,99	0,06	3,89	1,81
***tpk3*Δ**	1,17	0,66	2,76	1,88	3,03	2,13
***vhs1*Δ**	0,87	0,22	3,15	1,69	3,76	2,27
***yak1*Δ**	1,31	0,06	3,19	0,62	2,42	0,36
***ych1*Δ**	1,14	0,56	4,78	2,44	4,17	0,08
***yck1*Δ**	1,10	0,14	4,01	2,35	3,58	2,05
***yck2*Δ**	1,82	0,98	6,04	4,28	3,23	1,33
***yck3*Δ**	0,75	0,08	3,85	1,82	5,02	1,90
***ygk3*Δ**	0,89	0,02	2,99	0,50	3,35	0,47
***ypk1*Δ**	0,92	0,47	6,37	0,19	8,04	4,30
***ypk2*Δ**	1,19	0,76	7,30	7,81	5,07	3,31
***ypk3*Δ**	1,04	0,13	4,84	3,82	4,74	3,81
***YPL150W*Δ**	0,97	0,32	2,62	0,76	2,71	0,11
***yvh1*Δ**	1,70	0,82	5,51	0,73	3,77	2,24
