# Supplementary material for: Lipid Signaling via Pkh1/2 Regulates Fungal CO2 Sensing through the Kinase Sch9
Source: mBio. 2017 Jan 31;8(1):e02211-16. doi: 10.1128/mBio.02211-16 (PMC5263247; doi:10.1128/mBio.02211-16)
Supplement: TABLE S3 [file mbo001173162st3.pdf]

Table S3: Kinase candidate genes identified in mutant library screening

Gene	Function and screening behaviour
**Sc*TPD3***	ScTpd3 is a subunit of the protein phosphatase 2A complex regulating cell morphogenesis and transcription (5, 6). Mutants exhibit growth delay and transcription deficiency in 37°C. This was apparent in RNA expression analysis resulting in divergent expression of the housekeeping gene Sc*ACT1.* Increase of *tpd3Δ* Sc*NCE103* ^{CO2} expression (2.16 ± 0.65) was significant, but no kinase activity was reported so far.
**Sc*PTP1***	ScPtp1 is a poorly characterized phosphatase, which might be involved in the regulation of filamentation and was shown to dephosphorylate the isomerase and expression regulator ScFpr3 (7, 8). In addition to the ambiguous increase of *ptp1Δ* Sc*NCE103* ^{CO2} expression (2.07 ± 0.72), no kinase activity has been described so far.
**Sc*BUD32***	ScBud32 is a kinase of the EKC/KEOPS complex responsible for tRNA modification and telomere maintenance (9, 10). *bud32Δ* has a severe growth defect, especially at 37°C and in minimal medium, which interfered with our experiments (11). *bud32Δ* showed significantly increased Sc*NCE103* ^{CO2} expression (3.38 ± 0.79). However, Sc*NCE103* ^{air} expression was even more elevated compared to WT, which resulted in a high fold change between Sc*NCE103* ^{air} and Sc*NCE103* ^{CO2} of >10.
**Sc*PTK2***	ScPtk2 is a kinase involved in ion transport and spermine uptake (12, 13). Upon repetitive testing, Sc*NCE103* ^{CO2} increase of *ptk2Δ* was not significant (2.44 ± 2.09). Furthermore, no link to carbon-related functions has so far been reported.
