# Supplementary material for: Lipid Signaling via Pkh1/2 Regulates Fungal CO2 Sensing through the Kinase Sch9
Source: mBio. 2017 Jan 31;8(1):e02211-16. doi: 10.1128/mBio.02211-16 (PMC5263247; doi:10.1128/mBio.02211-16)
Supplement: TABLE S4 [file mbo001173162st4.pdf]

Table S4: Mean Sc*NCE103* expression and standard deviation (SD) of *S. cerevisiae SCH9* regulator mutants, phosphoablative mutants and *S. cerevisiae* WT under rapamycin treatment

	NCE103 ^{CO2}	**SD** ^{CO2}	***NCE103*** ^{air}	**SD** ^{air}
S. c. **WT + DMSO**	1,11	0,33	4,55	2,38
S. c. **WT + 200nM rapamycin**	1,85	0,46	15,26	5,65
**15 Dau**	0,86	0,15	2,75	0,41
pkh1 ^{ts} ***pkh2Δ***	1,93	0,32	18,58	1,30
sch9Δ + SCH9	1,41	0,22	4,28	0,97
sch9Δ + SCH9 ^{6A}	1,40	0,21	4,03	0,93
sch9Δ + SCH9 ^{T570A}	2,70	0,59	8,25	4,12
sch9Δ + SCH9 $^{T570A,\ 6A}$	2,92	1,11	7,72	1,65
