# Supplementary material for: Lipid Signaling via Pkh1/2 Regulates Fungal CO2 Sensing through the Kinase Sch9
Source: mBio. 2017 Jan 31;8(1):e02211-16. doi: 10.1128/mBio.02211-16 (PMC5263247; doi:10.1128/mBio.02211-16)
Supplement: TEXT S1 [file mbo001173162s1.pdf]

Supplemental methods

LC-MS/MS Analysis

To detect CO_2-dependent phosphorylation in ScCst6, Sc*CST6* overexpressing *S. cerevisiae* cultures were induced for 6 h in YEP with 2% galactose under 5% CO_2 at 30°C. Cells were harvested and ScCst6-His was purified using IMAC. For mass spectrometry analysis, in-gel digest with Trypsin-LysC, AspN, GluC (all Promega) or LysargiNase (14) and in-solution digest with Trypsin-LysC and GluC was performed. For in-gel digests, samples were applied to 4-20% SDS-PAGE, total protein was stained with Coomassie Blue and specific bands were cut out. After destaining, proteins were reduced with 25mM DTT and alkylated with 55 mM iodoacetamid in 25 mM NH_4HCO_3. For in-solution digest, IMAC eluates were reduced with 200mM tris(2-carboxyethyl)phosphine and alkylated with 375 mM iodoacetamid in 100 mM NH_4HCO_3. For Trypsin-LysC, AspN and LysargiNase 1.25 µg protease per digest were used at 37°C overnight. For GluC, 2.5µg protease + 0.5mM Glu-Glu peptide in 50mM NH_4HCO_3 were used. Peptides were extracted with trifluoracetic acid and increasing concentrations of acetonitrile (50-90%) for in-gel digest. Phosphopeptide enrichment was carried out using the TiO_2 method (TiO_2 Spin Tip Sample Prep Kit, protea, Morgantown, West Virginia) and remaining peptides were purified with C18 mini columns (C18 Spin Tip Sample Prep Kit, protea). Dried peptides were solubilized in MS buffer (0.05% trifluoracetic acid in 2% acetonitril/98% H_2O). LC-MS/MS analysis was carried out on an Ultimate 3000 nano RSLC system coupled to a QExactive Plus mass spectrometer (both Thermo Fisher Scientific). For separation of pre-concentrated peptides, an Acclaim Pep Map RSLC column (15 cm x 75 µm, 2 µm) was used as stationary phase (Thermo Fisher Scientific). The binary mobile phase consisting of eluent A (0.1% formic acid in H_2O) and eluent B (0.1% formic acid in 90/10 ACN/H_2O v/v) was applied for 135 min non-linear gradient elution. The Nanospray Flex Ion Source (Thermo Fisher Scientific) was used to generate positively charged ions at 2.2 kV spray voltage. The hybrid quadrupole/orbitrap mass analyser was operated in Full MS / dd MS^2 (TopN) mode at mass resolutions of 70K and 17.5K (FWHM), respectively, using stepped collision energies of 24, 30 and 36V.

Protein database search

Thermo raw files were processed via the Proteome Discoverer (PD) v1.4.0.288. Tandem mass spectra were searched against the NCBI protein database of *Saccharomyces cerevisiae* using the algorithms of Mascot v2.4.1 (Matrix Science), Sequest HT and MS Amanda. Two missed cleavages were allowed for all protease digestions. The precursor mass tolerance was set to 10 ppm and the fragment mass tolerance was set to 0.02 Da. At least 2 peptides per protein and a false discovery rate (FDR) <1% were required for positive protein hits. For calculation of the phosphosite probability the phospho*RS* 3.1 PD node has been used (15).
