# Supplementary material for: Lipid Signaling via Pkh1/2 Regulates Fungal CO2 Sensing through the Kinase Sch9
Source: mBio. 2017 Jan 31;8(1):e02211-16. doi: 10.1128/mBio.02211-16 (PMC5263247; doi:10.1128/mBio.02211-16)
Supplement: TEXT S2 [file mbo001173162s2.pdf]

Supplemental references

1. **Inagaki M, Schmelzle T, Yamaguchi K, Irie K, Hall MN, Matsumoto K.** 1999. PDK1 homologs activate the Pkc1-mitogen-activated protein kinase pathway in yeast. Mol Cell Biol 19:8344-8352.
2. **Kitada K, Yamaguchi E, Arisawa M.** 1995. Cloning of the *Candida glabrata TRP1* and *HIS3* genes, and construction of their disruptant strains by sequential integrative transformation. Gene 165:203-206.
3. **Gillum AM, Tsay EY, Kirsch DR.** 1984. Isolation of the *Candida albicans* gene for orotidine-5'-phosphate decarboxylase by complementation of *S. cerevisiae ura3* and *E. coli pyrF* mutations. Mol Gen Genet 198:179-182.
4. **Homann OR, Dea J, Noble SM, Johnson AD.** 2009. A phenotypic profile of the *Candida albicans* regulatory network. PLoS Genet 5:e1000783.
5. **van Zyl W, Huang W, Sneddon AA, Stark M, Camier S, Werner M, Marck C, Sentenac A, Broach JR.** 1992. Inactivation of the protein phosphatase 2A regulatory subunit A results in morphological and transcriptional defects in *Saccharomyces cerevisiae*. Mol Cell Biol 12:4946-4959.
6. **van Zyl WH, Wills N, Broach JR.** 1989. A general screen for mutant of *Saccharomyces cerevisiae* deficient in tRNA biosynthesis. Genetics 123:55-68.
7. **Fasolo J, Sboner A, Sun MG, Yu H, Chen R, Sharon D, Kim PM, Gerstein M, Snyder M.** 2011. Diverse protein kinase interactions identified by protein microarrays reveal novel connections between cellular processes. Genes Dev 25:767-778.
8. **Wilson LK, Benton BM, Zhou S, Thorner J, Martin GS.** 1995. The yeast immunophilin Fpr3 is a physiological substrate of the tyrosine-specific phosphoprotein phosphatase Ptp1. J Biol Chem 270:25185-25193.
9. **Downey M, Houlsworth R, Maringele L, Rollie A, Brehme M, Galicia S, Guillard S, Partington M, Zubko MK, Krogan NJ, Emili A, Greenblatt JF, Harrington L, Lydall D, Durocher D.** 2006. A genome-wide screen identifies the evolutionarily conserved KEOPS complex as a telomere regulator. Cell 124:1155-1168.
10. **Srinivasan M, Mehta P, Yu Y, Prugar E, Koonin EV, Karzai AW, Sternglanz R.** 2011. The highly conserved KEOPS/EKC complex is essential for a universal tRNA modification, t6A. EMBO J 30:873-881.
11. **Stocchetto S, Marin O, Carignani G, Pinna LA.** 1997. Biochemical evidence that *Saccharomyces cerevisiae YGR262c* gene, required for normal growth, encodes a novel Ser/Thr-specific protein kinase. FEBS Lett 414:171-175.
12. **Kaouass M, Audette M, Ramotar D, Verma S, De Montigny D, Gamache I, Torossian K, Poulin R.** 1997. The *STK2* gene, which encodes a putative Ser/Thr protein kinase, is required for high-affinity spermidine transport in *Saccharomyces cerevisiae*. Mol Cell Biol 17:2994-3004.
13. **Erez O, Kahana C.** 2002. Deletions of *SKY1* or *PTK2* in the *Saccharomyces cerevisiae trk1Δ trk2Δ*mutant cells exert dual effect on ion homeostasis. Biochem Biophys Res Commun 295:1142-1149.
14. **Huesgen PF, Lange PF, Rogers LD, Solis N, Eckhard U, Kleifeld O, Goulas T, Gomis-Ruth FX, Overall CM.** 2015. LysargiNase mirrors trypsin for protein C-terminal and methylation-site identification. Nat Methods 12:55-58.
15. **Taus T, Kocher T, Pichler P, Paschke C, Schmidt A, Henrich C, Mechtler K.** 2011. Universal and confident phosphorylation site localization using phosphoRS. J Proteome Res 10:5354-5362.
